# Analysis of Chimpanzee History Based on Genome Sequence Alignments

**DOI:** 10.1371/journal.pgen.1000057

**Published:** 2008-04-18

**Authors:** Jennifer L. Caswell, Swapan Mallick, Daniel J. Richter, Julie Neubauer, Christine Schirmer, Sante Gnerre, David Reich

**Affiliations:** 1 Department of Genetics, Harvard Medical School, Boston, Massachusetts, United States of America; 2 Department of Anthropology, Harvard College, Cambridge, Massachusetts, United States of America; 3 Broad Institute of Harvard and MIT, Cambridge, Massachusetts, United States of America; 4 Department of Molecular and Cell Biology, University of California Berkeley, Berkeley, California, United States of America; University of Oxford, United Kingdom

## Abstract

Population geneticists often study small numbers of carefully chosen loci, but it has become possible to obtain orders of magnitude for more data from overlaps of genome sequences. Here, we generate tens of millions of base pairs of multiple sequence alignments from combinations of three western chimpanzees, three central chimpanzees, an eastern chimpanzee, a bonobo, a human, an orangutan, and a macaque. Analysis provides a more precise understanding of demographic history than was previously available. We show that bonobos and common chimpanzees were separated ∼1,290,000 years ago, western and other common chimpanzees ∼510,000 years ago, and eastern and central chimpanzees at least 50,000 years ago. We infer that the central chimpanzee population size increased by at least a factor of 4 since its separation from western chimpanzees, while the western chimpanzee effective population size decreased. Surprisingly, in about one percent of the genome, the genetic relationships between humans, chimpanzees, and bonobos appear to be different from the species relationships. We used PCR-based resequencing to confirm 11 regions where chimpanzees and bonobos are not most closely related. Study of such loci should provide information about the period of time 5–7 million years ago when the ancestors of humans separated from those of the chimpanzees.

## Author Summary

Studies of population history traditionally examine a small number of genetic regions in many individuals; however, with genome sequencing technologies it is possible to assemble data sets with thousands more aligned sequences albeit in fewer individuals. To explore whether such data can provide useful insights about population history, we assembled large-scale data sets consisting of overlaps of random genome sequencing reads from chimpanzees and bonobos. Analysis of these data finds that bonobos and chimpanzees split from each other about 1.29 million years ago, western and central chimpanzees about 0.51 million years ago, and eastern and central chimpanzees at least 50,000 years ago. We find that the chimpanzee population has fluctuated significantly in size over the past half million years, with the central chimpanzee population size expanding dramatically, and the western chimpanzee population size contracting. Surprisingly, we also find that there are widespread regions of the genome where chimpanzees and bonobos are less closely related to each other than any of them are to humans. In these regions, chimpanzees and bonobos share a common genetic ancestor dating back to speciation from humans, providing a new source of information about that evolutionary event.

## Introduction

At least four distinct populations of chimpanzees have been defined based on morphological and geographic criteria, including bonobos (*Pan paniscus*) and three common chimpanzee populations: eastern (*Pan troglodytes schweinfurthii*), central (*Pan troglodytes troglodytes*), and western (*Pan troglodytes verus*) [1]. Genetic studies have confirmed the distinctiveness of the chimpanzee populations [2],[3],[4], and have also documented striking differences among them; for example, central chimpanzees harbor ∼2.5 times as much genetic variation as western chimpanzees, more than is observed in any human population [3],[5],[6],[7],[8],[9]. Allele frequency differentiation among some pairs of chimpanzee populations—for example western and central chimpanzees—is also known to be higher than between any pair of human populations [9].

In contrast with studies of human history—for which there is a rich fossil record that can complement and inform genetic studies—the dearth of chimpanzee fossils [10] means that nearly all information about chimpanzee demographic history must come from genetic data. The best current understanding of chimpanzee history comes from small collections of genomic loci amplified by polymerase chain reaction (PCR). The two largest data sets of this type were collected by Yu et al. [8], who studied ∼23 kilobases in 9 bonobos, 2 eastern, 5 central, and 6 western chimpanzees, and Fischer et al. [9], who studied ∼22 kilobases in 9 bonobos, 10 eastern, 10 central, and 10 western chimpanzees. Analyses of these data sets by fitting the data to an Isolation and Migration (IM) model have resulted in important inferences about chimpanzee history [11],[12]: that bonobos and common chimpanzees separated ∼1 million years ago (Mya); western and central chimpanzees separated ∼0.5 million Mya; there was a ∼3-fold expansion in the central chimpanzee population size since the western-central population separation; and there has been migration between western and central chimpanzees since they separated. While these analyses provide a baseline set of parameter estimates that can be used to understand the relationships among the chimpanzee populations, the estimates also have substantial uncertainty. We aimed to generate a new kind of data and a model for analyzing the data that would increase the precision of previous estimates and be sensitive to different features of demographic history.

We sequenced 26,495 reads from a bonobo (B) and 36,083 from an eastern chimpanzee (E), using a standard plasmid end-sequencing technique that obtains pairs of reads each about 800 base pairs in length (up to 1,600 base pairs when both ends of the clone are considered together) and separated by about 4 kilobases. We then combined these data with publicly available data from the chimpanzee and macaque genome projects: 1,193,115 reads from three central chimpanzees (C), 20,632,928 from three western chimpanzees (W), and 13,810,571 from macaque (M) [5],[13]. By aligning all reads to the human reference sequence (H) [14], we generated nine different data sets, defined by the combinations of samples in the alignments. The five-sequence data sets are designated C_1_C_2_WHM, W_1_W_2_CHM, CWBHM, and ECWHM, where letters are used to indicate the species that are included (for example, C_1_C_2_WHM denotes two central chimpanzees, a western chimpanzee, a human, and a macaque). The four-sequence alignments are designated C_1_C_2_WH, W_1_W_2_CH, CWBH, and ECWH, and the three-species alignment is designated WBH (Table 1). (We used the alignments of smaller numbers of individuals to obtain more precise estimates of certain demographic parameters.) These data sets contain much more alignment of chimpanzee sequence than have previously been available. For example, the CWBHM data set (598,814 bp) includes >20 times more alignment of central, western, and bonobo DNA than any population genetic data set studied to date [6],[8],[9].

**Table 1:** Genetic divergence between pairs of chimpanzee populations.

Base pairs (# regions)	5-group					4-group				3-group	Absolute time assuming 7 Mya human-chimp divergence and most precise estimate at left
	C_1_C_2_WHM	W_1_W_2_CHM	CWBHM	ECWHM	CBHOM*	C_1_C_2_WH	W_1_W_2_CH	CWBH	ECWH	chimp-bonobo-human	
	5,026,184 (21,810)	13,683,131 (52,294)	598,814 (2,688)	970,928 (4,192)	12,013,035 (18,985)	8,803,603 (35,670)	25,167,329 (98,258)	918,609 (3,755)	1,285,206 (5,249)	8,205,541 (19,674)	
**Pop. comparison**											
Central-Central	.154±.002 (.159±.002)					.157±.002					1.10±0.01 Mya
Western-Western		.062±.001 (.065±.001)					.064±.001				0.44±0.01 Mya
Central-Western	.166±.002 (.172±.002)	.168±.001 (.176±.002)	.155±.007 (.160±.007)	.161±.005 (.169±.005)		.172±.002	.174±.001	.168±.006	.167±.004		1.22±0.01 Mya
Eastern-Central				.151±.005 (.158±.005)					.158±.004		1.11±0.03 Mya
Eastern-Western				.165±.005 (.169±.005)					.175±.004		1.23±0.03 Mya
Chimp-Bonobo			.288±.009 (.307±.010)		0.322±.004 (.320±.004)			.311±.008		.314±.003	2.20±0.02 Mya

Notes: All data sets are restricted to the autosomes, and all estimates are given as a fraction of human-chimpanzee genetic divergence within the same alignment. For the 5-group alignments, we present in parentheses estimates correcting for recurrent mutation (calculated by EM analysis as described in Text S3).

*: The CBHOM data set adds to the 9 data sets that are the focus of most of this study. It consists of an alignment of chimpanzee-bonobo-human-orangutan-macaque, using the specialized alignment procedure described in Text S8. The estimate of chimpanzee-bonobo divergence is consistent with the other alignments.

The genome sequence alignments we used in our analysis are not only large, but also different in nature from traditional population genetics data sets. While genome sequence alignments have the advantage that they include orders of magnitude more alignment compared with traditional population genetic data sets (and are becoming increasingly practical to generate with new sequencing technologies), they have the disadvantage that only a few individuals are available for each region of alignment and so there is limited information about allele frequencies. A methodological question in population genetics is whether this different type of data can provide new information about history. Here, we demonstrate that genome sequence alignments can be used to provide insights about population history that are not accessible from the analysis of traditional, smaller data sets.

## Results

### Sequence Alignments and Data Quality Filtering

A challenge in studying overlaps of random genome sequences is that so much data is generated that it is impossible to curate the data manually. It is therefore crucial to develop a set of automated data quality filters that can be used to produce a data set with a very small base-calling error rate. As our goal was to obtain accurate allele calls, we were willing to lose a substantial fraction of our data set as long as what remained was reliable. Our requirements for a low error rate in the data were such that we could not simply use the published chimpanzee genome sequence as an input into our analysis: its error rate [5] was not sufficiently different from the rate of divergent sites among chimpanzees to allow us to be confident about divergent site identification. We turned instead to the raw data from the sequencing reads, and computationally implemented ten filters, using base quality as well as other information (*Materials and Methods*), to limit our analysis to the most reliable sequence (Table 1). In Table S1, we show that after application of our filters, we obtained stable estimates of key genetic parameters.

To empirically estimate the error rate in our data, we used a mass spectrometry based technology (Sequenom MassArray) [15] to genotype 467 of the divergent sites that we identified from the shotgun sequence alignments. Our genotyping panel of 6 bonobos, 7 eastern chimpanzees, 15 central chimpanzees, and 25 western chimpanzees included all of the samples that had been used in the genome sequence alignments. Only eight of the divergent sites did not give a perfect match between genotyping and sequencing, providing an upper bound on the discrepancy rate of (8 discrepancies)/((3 chimpanzees compared)×(467 divergent sites)) = 0.6% (Text S1).

To confirm that the samples used in the sequence alignments can be appropriately labeled as one bonobo, one eastern chimpanzee, three central chimpanzees, and three western chimpanzees, we also carried out principal components analysis (PCA) on the genotyping data (Text S2). This allowed us to characterize the relationships among the samples used in sequencing, and samples whose populations had previously been confirmed in a microsatellite-based analysis of population structure [4]. We found that all the samples in the sequence alignments are appropriately labeled. In particular, Clint, the captive-born chimpanzee used for the public genome sequence whose population origin had not been previously confirmed [5], falls squarely in the western chimpanzee cluster (Text S2). We conclude that Clint can be confidently treated as a western chimpanzee for population genetic studies.

### Genetic Measurements Are Consistent between Our Data and Previous Studies

Traditional population genetic data sets are based on small numbers of loci that are amplified by PCR and studied in multiple samples from each population of interest. By contrast, our data sets consist of large numbers of loci that are randomly distributed across the genome where “shotgun” sequencing reads happen to overlap. To verify that our data set was not systematically biased relative to data sets generated using PCR-based methods, we compared our estimates of genetic parameters with the largest chimpanzee population genetic variation data set published to date [9], which analyzed 9 bonobos, 10 eastern, 10 central, and 10 western chimpanzees over about 22 kb. For 15 measurements that we could make in both data sets, the estimates were consistent (all within 1.7 standard deviations) (Text S2). Further, our estimates of genetic parameters are much more precise (standard errors up to 12.8 times smaller; Text S2), suggesting that our data set may have power to resolve novel features of chimpanzee history.

### Central and Eastern Chimpanzees Are Most Closely Related in Time

Becquet et al. [4] published an analysis of microsatellite allele frequencies in bonobos, eastern, central, and western chimpanzees, which showed that western chimpanzees were the first of the common chimpanzees to separate, and that eastern and central chimpanzees are more closely related in time. Our data confirms this, showing that western chimpanzee genetic divergence from central chimpanzees is 1.10±0.03 times larger than central-eastern genetic divergence (similarly, western genetic divergence from eastern chimpanzees is 1.11±0.04 times larger) (Table S2). In what follows, we assume that eastern and central chimpanzees are most closely related in time, although we also consider the possibility of western-central migration continuing after the eastern-central population split.

### Estimates of Demographic Parameters for Bonobos, Central, and Western Chimpanzees

**Figure 1:**
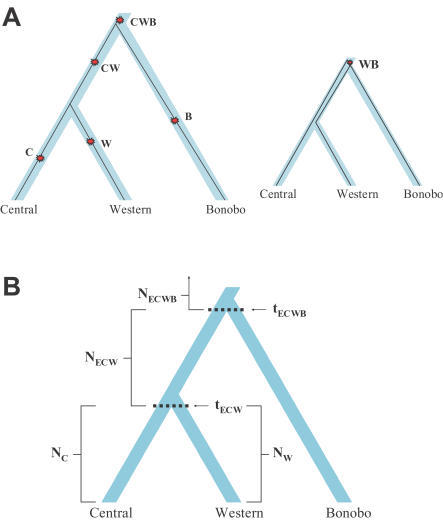
Schematic of our six-parameter model for analysis of the history of bonobos, central, and western chimpanzees. (A) Each five-group alignment has divergent site types that correspond to a branch in the tree: the lengths of branches are estimated from the observed numbers of the corresponding types of sites. The larger tree shows five possible types of sites (using the CWBH alignment as an example), and how they would be generated by single historical mutations. The smaller tree corresponds to one of the two rarer divergent site types that can arise when the genes from the two most closely related groups (central and western chimpanzee) share a common ancestor prior to the separation of the less closely related population (bonobo). (B) In the six-parameter model of chimpanzee evolution, the separation time of central chimpanzees and western chimpanzees is t_ECW_, the separation time of chimpanzees and bonobos is t_ECWB_, N_C_ and N_W_ specify the modern effective sizes of the central and western chimpanzee populations, and N_ECW_ and N_ECWB_ the effective sizes in the two earlier epochs. Although we do not include eastern chimpanzee in this analysis, the notations for t_ECW_, t_ECWB_, N_ECW_, and N_ECWB_ refer to eastern chimpanzee because eastern form a clade with central chimpanzees.

To translate the estimates of genetic divergence (Table 1) into inferences about history, we focused on three data sets: C_1_C_2_WHM, W_1_W_2_CHM, and CWBHM. Assuming that mutations have accumulated at a constant rate over time, these three data sets can be used to constrain the demographic history of chimpanzees. As a historical model, we assumed that chimpanzee populations have been freely mixing and of constant size over three epochs (later, we consider more complex models involving migration). The model has six parameters: t_ECW_ (time of separation of western chimpanzees from central and eastern), t_ECWB_ (time of separation of the common chimpanzees from bonobos), N_C_ (modern population size of central chimpanzees), N_W_ (modern population size of western chimpanzees), N_ECW_ (size of the chimpanzee population ancestral to central and western separation), and N_ECWB_ (size of the chimpanzee population ancestral to common chimpanzees and bonobos) (Figure 1).

To fit the data to the model, we began by confirming that divergent sites among the chimpanzees have accumulated at an approximately constant rate so that they can be used as a “molecular clock” to estimate elapsed time. We verified the reliability of this clock by using “rate tests” in which we compared all possible pairs of chimpanzee populations, and found no evidence that any has experienced an excess rate of mutations since its separation from the others (Table S3). Previous work suggests that the molecular clock assumption is reliable over an even longer time period, back at least to the human-chimpanzee split [16]. This increases our confidence that it can be used in the more conservative context of analyzing chimpanzee history. We next used an Expectation Maximization (EM) algorithm (Text S3) to convert the counts of divergent sites to estimates of time, correcting for the fact that a small fraction of the sites have been affected by recurrent mutation (Table S4; Table S5) [16]. This analysis has some similarities to previously described methods for correcting for recurrent mutation [16], but has the virtue of allowing genealogical trees to vary across loci.

Using the estimates of branch lengths that emerge from the EM analysis and fitting these to our model of demographic history using the procedure in Text S4, we estimate that common chimpanzees and bonobos separated 1.29 million years ago (Mya) (90% credible interval 1.14–1.46 Mya) and that central and western chimpanzees separated 0.51 Mya (0.43–0.58 Mya). These are in the range of previous inferences based on traditional genetic data, but the estimates have narrower credible intervals reflecting the larger size of our data set (Table 2). We caution that all these date estimates have systematic uncertainty as they are obtained by assuming that human-chimpanzee autosomal genetic divergence averaged 7 Mya [12] *(Materials and Methods*). The true genetic divergence time between humans and chimpanzee could be anywhere between 6–8 Mya [16] as the calibration to the fossil record is uncertain.

**Table 2:** Estimates of key parameters of chimpanzee history.

Parameter	Name	Our estimates of six parameters (90% credible interval)	Won and Hey [11] rescaled to use same calibrations* (90% credible interval)	Becquet and Przeworski [12] * (90% credible interval)
Central-Western pop. separation time	t_ECW_	0.51 Mya (0.43–0.59 Mya)	0.49 Mya (0.30–0.73 Mya)	0.44 Mya (0.32–1.10 Mya)
Chimp-Bonobo pop. separation time	t_ECWB_	1.29 Mya (1.14–1.45 Mya)	1.02 Mya (0.69–1.55 Mya)	0.90 Mya (0.68–1.17 Mya)
Central population size	N_C_	118,000 (91,000–159,000)	24,400 (17,200–35,600)	27,500 (18,300–59,700)
Western population size	N_W_	9,100 (8,100–10,000)	6,700 (4,600–9,400)	9,800 (7,700–12,900)
Central-Western ancestral pop. size	N_ECW_	16,000 (12,400–19,600)	4,600 (180–9,900)	15,000 (6,100–22,400)
Chimp-Bonobo ancestral pop. size	N_ECWB_	20,900 (16,400–25,500)	13,800 (26–25,900)	33,600 (25,200–46,800)

Note: Estimates of six parameters of demographic history with 90% credible intervals from bootstrap analysis (Text S4). Estimates of absolute ages (in years) and population sizes are all based on assuming that human-chimpanzee genetic divergence occurred 7 Mya, and assume 20 years per generation. For comparison, we also present estimates of the same parameters from two previous studies of chimpanzee history [11],[12]. The fact that the previous studies jointly estimated migration rate along with the other parameters means that our credible intervals are not fully comparable with the previous studies, which had to estimate more complex models. The only credible intervals that are fully appropriate to compare are those for t_ECWB_ and N_ECWB_, since we found that they are not substantially affected by assumptions about the central-western migration rate (Figure 2).

*: To make our estimates comparable to the other studies, we multiplied all the ages and population size estimates from the Won and Hey analysis [11] by 7/6 (since they used 6 rather than 7 Mya for human-chimpanzee genetic divergence), and further multiplied population sizes by 15/20 (since they assumed 15 not 20 years per generation). We did not rescale the estimates from the Becquet and Przeworski analysis [12], since they used the same estimate of 20 years per generation as we did, and assumed a mutation rate of μ = 1.0×10^−9^ per base pair per year, which corresponds to a calibration date of 7 Mya for human-chimpanzee genetic divergence and 0.0128 differences between these two species per base pair.

The estimates of population size changes—in contrast with the estimates of absolute time—are not affected by uncertainties arising from calibration to the fossil record. Under the model of demographic history in Figure 1 we infer that the effective population size of central chimpanzees has been N_C_/N_W_ = 12.9 (10.1–17.1) times larger than western chimpanzees since these populations split, while the western chimpanzee population size has decreased by a factor of ∼2-fold: N_W_/N_ECW_ = 0.44 (0.35–0.56) and N_W_/N_ECWB_ = 0.57 (0.41–0.79). A previous study found a hint of a western chimpanzee population contraction [6], but this was not significant in subsequent analyses [11],[12], probably because of the limited data set size.

Later in this study, we explore how complications to the demographic model, especially migration between western and central chimpanzees since initial population separation, would affect estimates of demographic parameters. While there are effects on some estimates, the novel inferences of a >4-fold expansion of the central chimpanzee population and a contraction in western history are robust to the presence of migration. A caveat is that we did not consider the possibility of substructure in the ancestral population of central and western chimpanzees prior to their separation. In the presence of such structure we would be overestimating the population size of the ancestors of central and western chimpanzees. Thus, the presence of ancestral structure would further strengthen the evidence of a large central chimpanzee population expansion, while weakening the evidence of a western chimpanzee population contraction.

### Reliability of the Procedure for Making Inferences about History from Shotgun Sequence Data

To check the reliability of our analysis, we used a coalescent computer simulation to generate synthetic data of the type that would be expected for our best fit model of the history of chimpanzees and bonobos (we simulated data using input parameters that corresponded to the parameters in Table 2). When we applied our analysis procedure to these simulated data, we found that we could accurately recover the correct demographic parameters. These results provide us with confidence that our analysis procedure is able to make reliable inferences about population history, at least when the history is similar to the model in Figure 1 (Text S5).

As a second check we repeated the analysis using a simplified model of history in which we assumed that N_ECW_ = N_ECWB_. This is a reasonable assumption since in Table 3 we estimate that N_ECW_/N_ECWB_ = 0.77 (0.51–1.18). With this simplification we can analyze the C_1_C_2_WHM and W_1_W_2_CHM data sets separately instead of modeling them and the CWBHM data simultaneously (Text S6). We now infer that the central effective population size has been 12.9 (10.1–17.2) times larger than the western effective population size, while the western population has changed by a factor of 0.53-fold (0.49–0.57), consistent with the results from the full six-parameter model. These analyses suggest that our analytical procedure extracts stable estimates of parameters of chimpanzee demographic history.

**Table 3:** Demographic estimates that are unaffected by calibrations to the fossil record.

	Explanation	Our estimate (90% credible interval)
N_C_/N_ECWB_	Ratio of modern central chimpanzee population size to ancestral chimpanzee-bonobo population size	5.7 (4.1–8.1)
N_C_/N_ECW_	Ratio of modern central chimpanzee population size to ancestral eastern-central-western chimpanzee population size	7.4 (4.9–11.6)
N_C_/N_W_	Ratio of modern central chimpanzee population size to modern western chimpanzee population size	12.9 (10.1–17.1)
N_W_/N_ECWB_	Ratio of modern western chimpanzee population size to ancestral chimpanzee-bonobo population size	0.44 (0.35–0.56)
N_W_/N_ECW_	Ratio of modern western chimpanzee population size to ancestral eastern-central-western population size	0.57 (0.41–0.79)
N_ECW_/N_ECWB_	Ratio of ancestral eastern-central-western population size to ancestral chimpanzee-bonobo population size	0.77 (0.51–1.18)
t_ECWB_/t_ECW_	Ratio of chimpanzee-bonobo population separation time to central-western chimpanzee population separation time	2.53 (2.07–3.19)
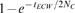	Probability of 2 central alleles coalescing more recently than the central-western chimpanzee population separation time	0.10 (0.08–0.13)
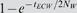	Probability of 2 western alleles coalescing more recently than the central -western chimpanzee population separation time	0.75 (0.73–0.77)
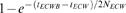	Probability of a central and western allele coalescing more recently than chimpanzee-bonobo population separation time	0.71 (0.66–0.75)

### Signal of Migration among Chimpanzee Populations

So far we have assumed a simple model of population history: a sudden split followed by no gene flow. In fact, evidence of western-central migration has been found in some [11],[12] although not all [4] previous studies. To test whether there is evidence of gene flow in our data set, we looked for asymmetries in the relationships among the populations.

We first tested for gene flow between western chimpanzees and central chimpanzees. This scenario is geographically plausible, since the Dahomey Gap between the western and central chimpanzees ranges is thought to have been bridged by forest as recently as ∼5,000 years ago [17], and two genetic analyses based on an Isolation and Migration model found evidence for such gene flow [11],[12]. Analyzing the ECWH(M) data sets, we searched for a very simple signal of gene flow: an excess of sites where central and western chimpanzees share an allele to the exclusion of eastern chimpanzee (CW) compared with sites where eastern and western chimpanzees share an allele to the exclusion of central chimpanzees (EW), or an excess of sites marking out eastern chimpanzees only (E) compared with central chimpanzees only (C). We observed excesses in the direction that would be expected from gene flow: CW/EW = 1.36±0.23 and E/C = 1.02±0.07 (ECWHM data), and CW/EW = 1.23±0.14 and E/C = 1.08±0.06 (ECWH data). To test whether the excesses were significant, we carried out a permutation test: in each of the clusters in our data set, we flipped the eastern and central chimpanzee labels with 50% probability. We repeated 10,000 such permutations, and recorded the proportion for which both the CW/EW ratio and E/C ratio (corrected for their correlation) were positive and the sum of their squares larger than we observed. We found asymmetry in both the ECWHM (P<0.03) and the ECWH data (P<0.02), weakly supporting the hypothesis of western-central gene flow.

We also tested for evidence of older gene flow between bonobos and common chimpanzees after initial separation of these populations. Under the hypothesis that all common chimpanzees are equally distantly related to bonobos, we would expect equal proportions of divergent sites clustering western chimpanzees and bonobos (WB) and central chimpanzees and bonobos (CB) in a CWBH(M) alignment. In the CWBHM data set, we observe WB/CB = 2.38±0.78, with 99.5% of bootstraps supporting a WB excess and western-bonobo gene flow. However, unlike the signal for central-western gene flow, this signal attenuates and is non-significant in the more inclusive CWBH data set (WB/CB = 1.20±0.28). Since no evidence for bonobo-western gene flow was found in previous studies [11],[12], and western chimpanzees today are geographically more separated from bonobos than either central or eastern chimpanzees, the evidence for such gene flow is marginal. While it will be important to explore this signal with further data, in what follows we do not consider how western-bonobo migration would affect our conclusions.

### Effect of Western-Central Migration on Inferences about Demographic History

**Figure 2:**
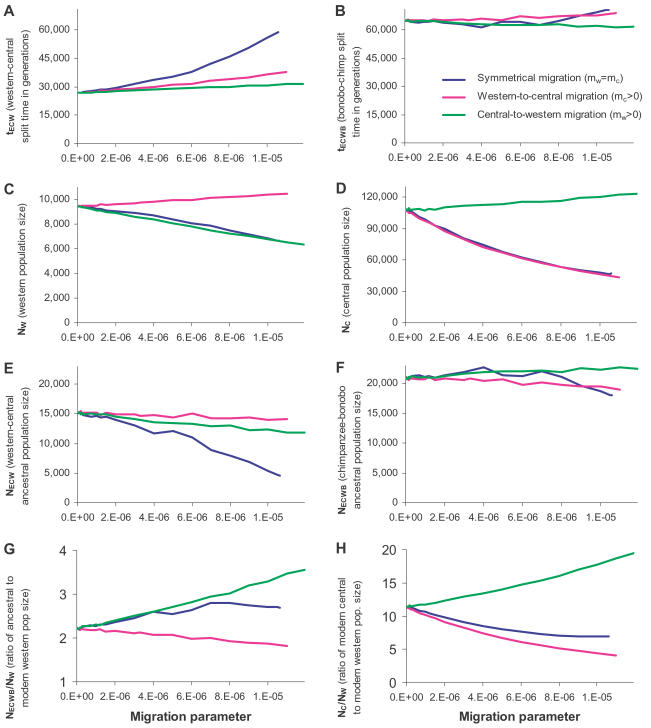
Inferred values of the six parameters of chimpanzee demographic history and key ratios of population sizes, for various assumptions about the migration rate per generation between western and central chimpanzees since the western-central population split. All plots consider the full range of migration rates for which we could obtain a reasonable fit to the data (Text S7), with the values matching those in Tables 2 and 3 for the zero-migration rate scenarios. (A) In the presence of migration, central-western population separation time t_ECW_ increases relative to the zero-migration scenario, but (B) the bonobo-common chimpanzee separation time estimate t_ECWB_ is unchanged. (C,D) Migration rate has a variable effect on our estimates of western and central population size, depending on the direction of the migration, but (E) increasing migration rate always decreases our estimate of N_ECW_, and (F) our estimate of ancestral bonobo-chimpanzee population size is unaffected by migration rate assumptions. (G) For all migration rates consistent with the data, we infer that the western population size contracted relative to the ancestral size by at least 1.8-fold (panel f divided by e), and (H) that the ratio of central to western size has been >4.1 (d divided by e).

Our test for migration as well as previous studies [11],[12] suggests the possibility of western-central gene flow since their initial population separation. To explore how this could affect our inferences about demographic parameters (Table 2), we first note that unlike previous Isolation and Migration (IM) analyses that take advantage of variation in genealogies across loci, our analysis is not very sensitive to migration; indeed, at P = 0.03 we barely detect a signal of migration at all. Rather than jointly estimating the migration rate and other demographic parameters in the face of this difficulty, we therefore considered the full range of migration rates consistent with the data and studied how estimates of demographic parameters are affected (Figure 2).

To accommodate the possibility of western-central migration, we added two additional parameters to our six-parameter model (Figure 2). We allowed there to be migration between western and central chimpanzees, with m_C_ designating the fraction of central chimpanzee genes replaced by western chimpanzee genes every generation and m_W_ designating the reverse direction of migration (these choices allow comparison to the IM model analyzed in refs. 11 and 12). Addition of migration into our model meant that we could no longer use the same analytical procedure for estimating demographic parameters. We therefore developed an alternative numerical procedure to obtain the divergent site rates expected under each migration model, and wrote software that iteratively searches for combinations of the six demographic parameters that provide the best fit to the data (Text S7). The method produced appropriate estimates of parameters when we tested it by simulation (Text S7). Since the new method is much slower than the method we developed not accommodating migration (Text S4), we do not present bootstrap credible intervals on the estimates that emerge from this method. However, we expect that conditional on knowing the migration rates the credible intervals would be similar in magnitude to those in Tables 2–3.

While migration affects parameter estimates (Figure 2), several inferences are robust to assumptions about migration. First, the t_ECWB_ and N_ECWB_ estimates are nearly unaffected by the assumed western-central migration rate, reflecting the fact that the ancestral population of bonobos and chimpanzees lived so long ago that more recent migration does not affect inferences about that population's history. Second, whatever migration rate we assume, the western population size N_W_ is inferred to have contracted by at least 1.8-fold compared with the long-term ancestral population N_ECWB_ size (Figure 2G). Third, we infer that the central chimpanzee population N_C_ was >4.1 times larger than western chimpanzees N_W_ since their split (Figure 2H) with most migration rate assumptions indicating more than an order of magnitude difference.

Our result that the central chimpanzee effective population size expanded by about an order of magnitude over the last half million years is especially interesting. We can obtain a best-estimate of the magnitude of this expansion, incorporating the possibility of migration, by combining results from IM analysis, which is particularly good at estimating migration rates, and our analysis, which is better at estimating population size changes because of the more precise estimates of genetic divergence parameters. Won and Hey [11] inferred a central chimpanzee expansion factor of N_C_/N_W_ = 3.6 using an IM model that also inferred western-to-central chimpanzee migration of 2N_C_m_C_ = 0.514 and 2N_W_m_W_ = 0. If we use the same migration parameters as Won and Hey (translating to m_C_ = 0.000033 and m_W_ = 0), and determine the central chimpanzee expansion factor from Figure 2H, we infer a much larger expansion factor than they estimated: N_C_/N_W_ = 8.

These results illustrate how combining analyses of traditional data sets and genome sequence alignments allows us obtain more information about population history than would be possible with either analysis alone. Simultaneously, the results highlight a discrepancy between the estimated size of the central expansion from genome sequence alignments and previous estimates. A possible explanation is statistical uncertainty due to limited data size in the earlier studies (Table 2). Another possibility is that IM methods to date have not taken into account the possibility of variability in mutation rates over time across loci [11],[12] whereas our analysis is not expected to be biased by this variability. As larger chimpanzee variation data sets are gathered and analyzed, it will be valuable to compare the results of IM analyses and the results using our methodology to assess whether evidence for a discrepancy persists.

### Combining Traditional Data with Genomic Alignments to Learn about Eastern and Bonobo History

While our data sets include up to two sequences from western and central chimpanzees, they include at most one sequence from bonobo and from eastern chimpanzee, and so our ability to make inferences about these populations is poorer. To obtain more insight about the history of the eastern and bonobo populations, we carried out an analysis in which we assumed that the six parameters in Table 2 are correct, and then combined these estimates with statistics from the recently published resequencing data set of Fischer et al. [9].

The measurements we obtained from the resequencing data set were: (i) allele frequency differentiation (F_ST_) between eastern and central chimpanzees [18], (ii) the ratio of genetic diversity within bonobos to genetic diversity within central chimpanzees, (iii) the ratio of genetic diversity within eastern chimpanzees to genetic fddiversity within central chimpanzees, (iv) the average heterozygosity of the single nucleotide polymorphisms (SNPs) discovered as polymorphic in nine bonobos, and (v) the average heterozygosity of SNPs discovered as polymorphic in ten eastern chimpanzees. All estimates of statistical error for genetic parameters were obtained by a weighted jackknife analysis for the Fischer et al. data (Text S2) just as they were for the shotgun sequence alignments [19].

**Figure 3:**
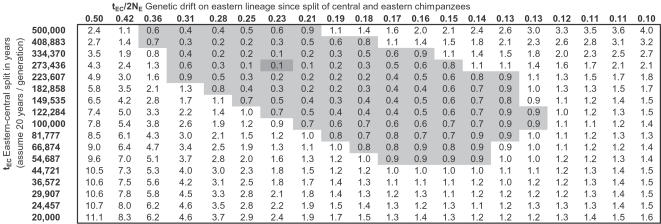
Inferences about parameters of eastern chimpanzee history. We ran our computer simulation of chimpanzee history using the six demographic parameters that were the best fits to our data under the assumption of no western-central migration (Table 2). We then varied the time t_EC_ of eastern-central separation and the modern eastern population size N_E_, exploring the full range of parameters consistent with three statistics of interest that we measured using data from ref. [9]: the F_ST_ value between eastern and central chimpanzees, the ratio of eastern to central chimpanzee genetic diversity, and the average heterozygosity of SNPs discovered as polymorphic within ten eastern chimpanzees. We found an excellent fit to our data for the parameters N_E_ = 30,078 and t_EC_ = 13,672 generations (∼273,000 years assuming 20 years per generation). The values in the cells give -log_10_ of the P-value for a χ^2^ statistic with three degrees of freedom. We indicate the 95% credible interval (gray) as the region where this is within 0.86 of the maximum (a likelihood ratio test, which is only approximate since the three statistics that we use to assess the fit are not fully independent). This analysis implies that, with approximately 95% probability, eastern and central chimpanzees split at least 50,000 years ago.

To explore whether there are combinations of demographic parameters for eastern chimpanzees that fit both data sets, we modified our simulation (Text S5) to allow eastern and central chimpanzees to split at time t_EC_. We forced the six parameters in Table 2 into the simulation, and varied N_E_ (eastern population size since separation from central) and t_EC_ (eastern-central separation time in generations, required to be <t_ECW_), searching for values of the parameters that provided the best fit to the data. Averaging over 50,000 replicates of each simulation, we calculated the quantity (observed-simulated)/(standard deviation of observed value) for statistics i, iii, and v, and then used the sum of the squares as an approximate chi-square statistic to evaluate the fit. As shown in Figure 3, the fit is excellent, and the maximum likelihood is that eastern and central chimpanzees split 273,000 years ago, with >95% confidence of a split >50,000 years ago using an approximate likelihood ratio test. In the presence of migration after the initial split of central and eastern chimpanzees, the population separation time would have to be even older to produce the degree of allele frequency differentiation that we observe.

Applying the same procedure to bonobos and varying N_B_ (the modern bonobo population size), we were not able to obtain a satisfactory fit to the data. The best fit infers N_B_ ∼16,000, but this is a poor match to the data (P = 0.016 from a chi-square distribution with 2 deg of freedom, summing the squares of [observed-simulated]/[standard deviation of observed data] for statistics ii and iv above). The reason for the poor fit to the bonobo data is that the average heterozygosity of SNPs discovered by resequencing nine bonobos (0.251±0.015 in the Fischer resequencing data [9]) is less than the 0.282 expected for a constant-sized population for this number of samples (P = 0.023). Since the bonobo-chimpanzee population separation occurred so long ago, the demography of the population ancestral to the bonobo-chimpanzee split has at most subtle effects on the allele frequency distribution today, and our analysis is essentially exploring whether the observed data can be fit by the assumption of a constant-sized population. Since we reject the model that the bonobo population has been constant in size, this analysis suggests more recent expansion in the history of bonobos, or alternatively population subdivision.

### Prediction of Incomplete Lineage Sorting Comparing Chimpanzees, Bonobos, and Humans

Our best estimates of bonobo-common chimpanzee population separation parameters (t_ECWB_ = 1.29 Mya and N_ECWB_ = 20,900) (Table 2) allow us to predict that there will be a substantial fraction of the genome in which chimpanzees and bonobos will be less closely related to each other than one of them is to human. In other words, the gene tree will be incongruent with the species tree in these regions, a phenomenon known as “incomplete lineage sorting”. Assuming for the sake of argument that human-chimpanzee speciation occurred 5.4 Mya (consistent with the estimates of ref. 16) and that the ancestral population of chimpanzees was effectively constant in size between that time and t_ECWB_, then the probability at any locus that bonobos and common chimpanzees will be unrelated all the way back to the time of human-chimpanzee speciation is 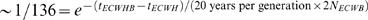. Thus, a prediction of our model is that in a few tens of megabases of the genome, there will be incomplete lineage sorting among the species.

### Empirical Confirmation of Incomplete Lineage Sorting

To evaluate the evidence for incomplete lineage sorting, we generated a new data set consisting of about 12 million base pairs of aligned sequence of chimpanzee, bonobo, human, orangutan, and macaque (CBHOM) (Text S8). The CBHOM data set includes two outgroups (both orangutan and macaque) helping to distinguish between divergent sites that arose due to incomplete lineage sorting and those that arose due to multiple mutations.

**Figure 4:**
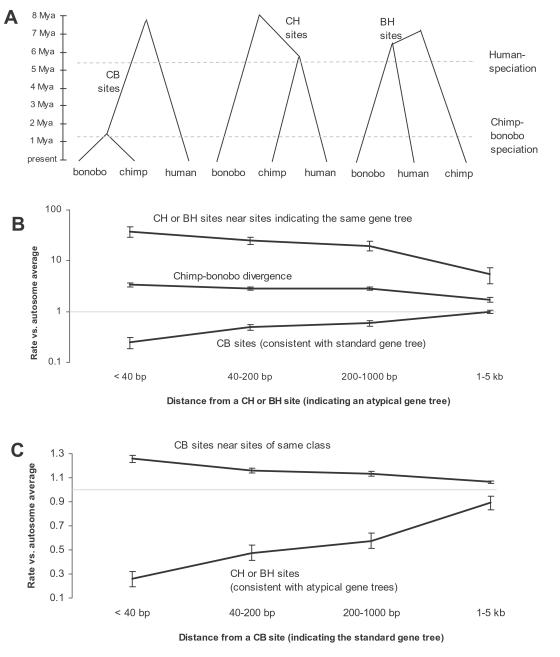
Genealogical trees relating chimpanzee, bonobo, human, orangutan, and macaque (CBHOM) vary in shape across the genome. (A) The most common is on the left, but if there is incomplete lineage sorting, different tree topologies such as the two on the right can occur. These should be detectable from CH sites that cluster chimpanzee and human to the exclusion of the other species, or BH sites that cluster bonobo and human. (B) In the subset of the CBHOM data that is within 40 base pairs of a CH or BH site, we observe a ∼38-fold excess of sites of the same class, a deficiency in CB sites typical of the standard genealogy (∼25% of the average), and a ∼3.3-fold excess of sites that differ between chimpanzee and bonobo, exactly as would be expected if in these regions, chimpanzees and bonobos are not most closely related and only share a common ancestor prior to human-chimpanzee speciation. (C) By contrast, very near to CB sites typical of the standard genealogy, we observe few CH or BH sites (∼26% of the genome average). These results confirm that the majority (∼74%) of CH and BH sites are marking out genuine regions where the genealogy is different from the species relationships. All bars in this figure correspond to one standard error.

The first line of evidence for incomplete lineage sorting in this data is the presence of 238 CH sites, where chimpanzees and humans cluster to the exclusion of the other primates, and 215 BH sites, where bonobos and humans cluster (Table 4; Figure 4A). To determine whether there are more CH and BH sites than would be expected due to recurrent mutation, we applied an expectation maximization (EM) analysis to the counts from Table 4. We observe a ∼3-fold excess of observed CH and BH sites over what would be expected if there was no incomplete lineage sorting but only recurrent mutation (χ^2^ = 673). If we instead allow for incomplete lineage sorting, we obtain an excellent fit to the counts (χ^2^ = 9) (Table 4). The EM analysis estimates that 73 percent of the CH sites and 70 percent of the BH sites in the CBHOM data set occur in regions of incomplete lineage sorting (Table 4).

**Table 4:** Evidence for chimp-bonobo-human incomplete lineage sorting.

Class*	Pattern	Observed	Expected in all data†		Alignments with strong CH clustering‡						Alignments with strong BH clustering‡				
			Allow incomplete lineage sorting	Only CB trees	#1538	#467	#1883	#473	#528	#148	#547	#518	#1298	#711	#678
**n_C_**	10000	6,425	6,434	6,543	1	2	2	2	1	3	1	2	2	7	3
**n_B_**	01000	6,520	6,512	6,628	3	7	2	2	6	6	1	2	0	2	3
**n_H_**	00100	20,432	20,408	20,397	1	3	0	2	3	0	4	0	1	0	3
**n_CB_**	11000	14,553	14,574	14,484	0	0	0	0	0	0	0	0	0	0	0
**n_CH_**	10100	237	238 (27% recurrent mut.)	75	4	2	3	3	2	1	0	0	0	0	0
**n_BH_**	01100	215	215 (30% recurrent mut.)	74	0	0	0	0	0	0	4	3	3	1	2
**n_CBH_**	11100	34,953	34,952	35,155	4	1	1	1	2	1	2	2	1	3	1
**n_O_**	00010	57,921	57,940	57,863	4	4	1	5	3	3	5	7	4	5	4
**n_M_**	11110	172,622	172,604	172,363	9	4	5	11	16	17	10	5	11	10	14
**n_CO_**	10010	76	72	79	0	0	0	0	0	0	0	0	0	0	0
**n_BO_**	01010	85	74	80	0	0	0	0	0	0	0	0	0	0	0
**n_HO_**	00110	716	673	770	0	0	0	0	0	0	0	0	0	0	0
**n_CBO_**	11010	752	798	916	0	0	0	0	0	0	0	0	0	0	0
**n_CHO_**	10110	190	208	242	0	0	0	0	0	0	0	0	0	0	0
**n_BHO_**	01110	211	206	239	0	0	0	0	0	0	0	1	0	1	0

*: Each divergent site class is designated by a string of 0's and 1's, the bases seen in chimp/bonobo/human/orangutan/macaque. The macaque allele is defined as state “0”.

**†:** Under a model of incomplete lineage sorting, our EM analysis (Text S3) obtains a good fit between observed and expected, with nominal χ^2^ = 9, and a prediction that 27% of CH sites and 30% of BH sites are due to recurrent mutation. These results are concordant with our observation (Fig. 4) that very close to CB sites, the rate of CH and BH sites is reduced to 26±6% of the average. If we only allow a model with genealogies clustering chimpanzees and bonobos, the best fit has a nominal χ^2^ = 673.

**‡:** We examined all 18,985 alignments, looking for ones where genealogical trees clustering CH or BH are favored over those clustering CB (>20,000:1 likelihood ratio), and in which the counts fit the proposed genealogies well (Text S8). Although these alignments should be treated with caution as they are extremes from a distribution, they are strong prospects for loci where chimpanzees and bonobos not being most closely related (alignment details are at genepath.med.harvard.edu/∼reich/Data%20Sets.htm).

**Figure 5:**
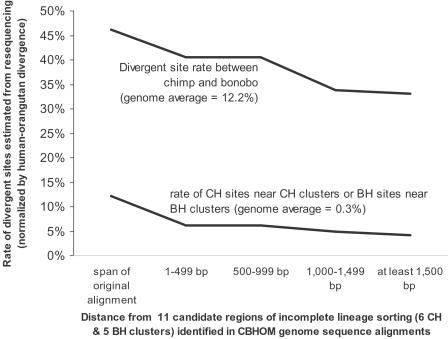
To validate regions of incomplete lineage sorting, we carried out laboratory-based follow-up of 11 regions where our main analysis found strong evidence in favor of a genealogy where chimpanzees and bonobos are not most closely related (likelihood ratio of >20,000:1). We targeted up to 5 kb centered on each of these regions for PCR-based resequencing, and only analyzed divergent sites that were independent of those found in the shotgun analysis (Text S9). We found an excess of CH sites and BH sites in regions previously identified as clustering these pairs of species (∼22 times the genome average), as would be expected if these regions have the genealogies inferred in Table 4. Chimpanzee-bonobo genetic divergence divided by human-orangutan genetic divergence is 38.4%, about three times the observed genome-wide rate of 12.2%, as expected if chimpanzees and bonobos share a common ancestor so long ago that it occurred prior to human-chimpanzee speciation. Both patterns attenuate with distance, as expected if the genealogies cover only a limited physical distance span.

The second line of evidence for incomplete lineage sorting is obtained by studying the regions close to CH and BH sites (Figures 4 and 5). Here, we observe a 38±9 fold excess of sites of the same class compared to the genome average, a reduction in the rate of CB sites, signatures of the standard genealogy, to 0.26±0.06 of the average, and a 3.4±0.4-fold elevation in chimpanzee-bonobo genetic divergence. These patterns reflect what we would expect if CH and BH sites mark out regions of incomplete lineage sorting. In such regions, chimpanzees and bonobos should share a genetic ancestor prior to human-chimpanzee speciation, explaining the great excess of genetic divergence in these regions compared with the genome-wide average.

The third line of evidence for incomplete lineage sorting comes from laboratory validation. We identified regions of the genome for follow-up validation by examining each of the 18,985 alignments in the CBHOM data in turn, and calculating a likelihood ratio that it was drawn from an atypical genealogy versus a region where chimpanzees and bonobos were most closely related (Text S8). We thus identified 11 candidate regions with strong evidence for unusual genealogies (likelihood ratio of >20,000:1; Text S8). To confirm these regions in our laboratory, we used PCR amplification followed by bidirectional sequencing to obtain up to 5 kb of sequence centered on each of the 11 regions (Text S9). Among the divergent sites that we identified in these regions, we observed a 22-fold excess of sites supporting the hypothesized genealogies, and a chimpanzee-bonobo genetic divergence that is more than 3 times the genome average (Table 5). Both patterns attenuate with distance as expected if the underlying genealogies have a limited physical span in the genome.

**Table 5:** Validation of 11 candidate regions of chimp-bonobo-human incomplete lineage sorting.

Region ID	Cluster pattern inferred from shotgun analysis	Chrom-osome	Position in Mb (Build36)	Bases targeted for resequencing	C	B	H	CB	CH	BH	O	Rate of CH sites in CH regions (and BH sites in BH regions)/human-orangutan genetic divergence *	Chimpanzee-bonobo genetic divergence/human-orangutan genetic divergence *
**528**	CH	1	52.3	4,333	13.5	14.8	13.2	12.4	3.3	1.3	59.8	4%	43%
**1538**	CH	1	193.2	3,717	11.1	15.0	12.7	9.0	4.0	0.0	64.0	5%	37%
**1883**	CH	1	231.4	4,647	6.7	10.1	11.2	8.9	2.8	1.3	80.3	3%	22%
**473**	CH	10	67.8	4,354	21.0	26.3	22.8	11.0	13.3	9.0	53.5	13%	71%
**148**	CH	13	39.0	4,374	6.4	8.4	16.4	19.2	4.0	0.0	73.4	4%	20%
**467**	CH	15	85.3	4,371	7.9	7.5	12.7	16.0	3.0	1.0	63.9	4%	24%
**547**	BH	1	55.0	4,366	11.5	8.5	13.8	20.6	1.0	2.0	47.3	3%	36%
**711**	BH	2	78.0	3,860	15.0	16.3	13.7	8.8	2.0	3.0	76.1	3%	38%
**678**	BH	4	86.4	4,373	20.6	9.6	16.5	7.8	0.4	4.4	54.5	6%	46%
**1298 ** †	BH	6	143.4	4,361	6.0	2.0	1.8	1.0	1.0	2.0	14.5	10%	57%
**518**	BH	14	78.5	4,336	20.5	6.5	13.2	6.1	0.0	12.8	71.5	13%	41%

Note: Results of resequencing of 11 candidate regions of incomplete lineage sorting. We targeted up to 5 kb for resequencing in 4 chimpanzees, 3 bonobos, 3 humans, and 3 orangutans. Divergent sites were filtered to remove sites that overlapped with the ones used to discoverer the regions (Text S9). The counts of the 7 possible classes of divergent sites can be non-integer due to within-species polymorphism.

*: We observe an excess of CH sites in regions where chimpanzees and humans cluster, and BH sites in regions where bonobos and humans cluster (6.6% vs. the genome-wide average of 0.3%). Chimpanzee-bonobo genetic divergence in these regions is also inflated: 38.4% on average versus the average of 12.2%.

**†:** Resequencing of region 1298 provided substantially less data than the other 10 regions. Nevertheless, the data showed a substantial excess of BH (n = 6) over CH (n = 2) sites at this locus, supporting the presence of incomplete lineage sorting.

Finally, we checked that the presence of regions of incomplete lineage sorting does not bias our inferences about chimpanzee and bonobo demographic history (Tables 2–3). We estimate that only about 33 of the 26,223 divergent sites in the CWBHM data set will be mislabeled due to regions where chimpanzees and bonobos are not most closely related (calculation not shown), a small proportion that we expect would only mildly affect our conclusions. We also explicitly carried out computer simulations of our inference procedure that include the phenomenon of incomplete lineage sorting (Text S5), and found that our conclusions about history are not appreciably biased by the presence of unusual genealogies.

## Discussion

We have generated large numbers of multiple sequence alignments from western chimpanzees, central chimpanzees, eastern chimpanzees, and bonobos. These alignments provide orders of magnitude more genetic data for studying the history of these populations than has been previously available. By analyzing these alignments, we have made inferences about history that are generally consistent with previous studies (Table 2), but are also qualitatively new because of our larger data sets. Our analyses suggest that the sizes of chimpanzee populations have varied over a larger dynamic range than was previously believed. We estimate that for central chimpanzees, the effective population size for the half million years since the split from western chimpanzees has been much larger than previous estimates [11],[12], probably greater than 100,000 (Table 2). We also obtain meaningful genetic results about the separation time of central and eastern chimpanzees, with our analyses suggesting that these populations separated >50,000 years ago.

These findings are also interesting in the context of geological history. The formation of the Congo River ∼1.5–2 Mya [20] is hypothesized to have been the event that separated the ancestors of bonobos (south of the river) from those of common chimpanzees (to the north) [21]. Our analysis suggests a population separation of 1.29 Mya (1.14–1.45), which is consistent with but more precise than previous estimates of 1.02 Mya (0.69–1.54) [11] and 0.90 Mya (0.68–1.17) (Table 2). If we had instead used an 8 Mya rather than 7 Mya calibration for human-chimpanzee genetic divergence—within the range of dates consistent with the fossil record—the upper end of two of these credible intervals would have overlapped the geological date. Thus, the genetic data can not rule out the hypothesis that the formation of the Congo River led to chimpanzee-bonobo speciation [20].

This study finally shows that there are widespread regions of the genome where the genealogies relating our closest living relatives are not the same as the species relationships. At these loci, chimpanzees and bonobos trace their ancestry independently to the time before speciation from humans. These regions are interesting because they may provide information about the period 5–7 million years ago when human and chimpanzee ancestors separated. A recent comparison of the genomes of humans, chimpanzees, and gorillas suggested that human-chimpanzee speciation was complex, possibly involving gene flow after initial population separation [16]. However, that study was not able to discern whether the complexity occurred on the human or chimpanzee side of the genealogy (or both) [16]. The presence of loci where chimpanzees and bonobos trace their ancestry independently back to that time should provide information about the side of the genealogy on which complex speciation occurred. It will be possible to access this rich source of information once a whole-genome sequence alignment of chimpanzee, bonobo, human, gorilla, and more distantly related primates becomes available.

## Materials and Methods

### DNA Sequence Alignments

We sequenced random fragments of the genome from a bonobo and an eastern chimpanzee using a plasmid end-sequencing technique (Table 1). These segments were aligned to the NCBI Build 34 human genome assembly using the same method described in ref. 16, and compared with previously generated large collections of sequencing reads from three central chimpanzees, three western chimpanzees, and a macaque that we aligned to the human genome assembly (Table S6). We aligned the sequences from each region using the Multiple Alignment Program [22] with parameters gap_size = 5, gap_open = 4, gap_extend = 3, match = 1, mismatch = −2. To ensure that only a single haplotype was sampled from each individual, we used only the single read containing the most contiguous aligned bases for each of the chimpanzee groups. In this way we assembled four five-group alignments: C_1_C_2_WHM, W_1_W_2_CHM, CWBHM, and ECWHM (Table 1). We also assembled four four-group alignments (C_1_C_2_WH, W_1_W_2_CH, CWBH, and ECWH), and one three-group alignment (WBH). The purpose of the alignments of smaller numbers of individuals was to include more data to estimate particular quantities of interest.

### Data Curation and Quality Filters

We filtered the DNA sequence alignments according to ten criteria designed to eliminate regions and bases of erroneous alignment or poor quality. The filters excluded: (1) bases that did not meet minimum quality restrictions, (2) bases inside or within two bases of a low-complexity region, (3) alignments that contained <100bp from all groups, (4) alignments in which one of the groups exhibited an unusually high rate of heterozygosity, (5) alignments where an unusually high number of reads from one group mapped to the same locus, (6) alignments where there was evidence of a significantly high accumulation of mutations on one part of the tree compared to the others, (7) alignments that mapped to known segmental duplications in humans or chimpanzees, (8) divergent sites that were adjacent to other divergent sites, (9) divergent sites overlapping CpG dinucleotides, which are known to be hypermutable, and (10) divergent sites with greater than two alleles across all five groups. Further details of these filters are provided in Text S10.

### Genotyping Data

To confirm the quality of our sequence alignments and confirm that “Clint” (the chimpanzee that was the focus of the chimpanzee genome project [5]) can be appropriately treated as being from the western population, we genotyped selected divergent sites discovered in 6 bonobos, 7 eastern chimpanzees, 15 eastern chimpanzees, and 25 western chimpanzees. These samples were largely a subset of those we analyzed in a study of chimpanzee population structure [4], but were supplemented to also include the 1 bonobo, 1 eastern, 3 central, and 3 western chimpanzees used in our alignments. Details of this genotyping and analysis of the data are provided in Text S1.

### Analytical Procedures

The analytical procedures used for our main estimates of demographic parameters are described in Notes S3 and S6. The computer simulations used to test the reliability of our inferences are described in Text S5. The expectation maximization (EM) algorithm used to estimate branchlengths in the presence of recurrent mutation is described in Text S3. The numerical procedure used for inferring demographic parameters in the presence of migration is described in Text S7. To convert our genetic estimates into population separation times and population sizes, we assumed 7 Mya for human-chimpanzee genetic divergence and 20 years per generation [6],[23].

### Evidence of Incomplete Lineage Sorting from Correlation in Divergent Site Rates in CBHOM Data

To study the rates of divergent sites as a function of distance from CB, CH, BH, HO, and CBO sites, we flagged all sites that were within a specified physical distance window of at least one such site, studying four distance windows of 2–39 bp, 40–199 bp, 200–999 bp, and 1–5 kb around each class of sites. Rates of each divergent site were then compared with the genome-wide average, and some of the most interesting results are presented in Figure 4. Standard errors were obtained by dividing the genome into 100 non-overlapping bins, leaving these bins out in turn to study the variability in the underlying rate estimates (standard errors from jackknife analysis).

### Resequencing To Confirm Putative Regions of Incomplete Lineage Sorting

Polymerase chain reaction (PCR) primers were tiled across 5 kilobases centered on 11 putative regions of incomplete lineage sorting. Bidirectional sequencing of amplicons of up to 500 base pairs was carried out using ABI 3730 sequencing. Divergent sites were identified using the SNP Compare software, which combines information from the Polyphred 5.0 software [24] and the PolyDHAN software (Richter et al. unpublished data). Further details about the generation and analysis of these data are provided in Text S10.

### Online Resources

The sequencing data we generated from eastern chimpanzees and bonobos are available from the NCBI trace archive: http://www.ncbi.nlm.nih.gov/Traces/trace.cgi. The sequence alignments and filtered data sets are available at our website: http://genepath.med.harvard.edu/∼reich/Data%20Sets.htm.

## Supporting Information

Table S1 Stability of inferences using different data filters
(0.06 MB DOC)

Table S2 Comparisons of genetic divergences
(0.04 MB DOC)

Table S3 “Rate tests” of the chimpanzee molecular clock
(0.04 MB DOC)

Table S4 A paucity of sites due to recurrent mutation
(0.04 MB DOC)

Table S5 Counts of divergent sites from 5-sequence alignments
(0.09 MB DOC)

Table S6 Sequencing reads available for analysis (prior to filtering)
(0.04 MB DOC)

Text S1 Validation of divergent sites discovered in the genome sequence alignments
(0.07 MB DOC)

Text S2 Consistency of our data with Fischer et al.
(0.07 MB DOC)

Text S3 Expectation Maximization (EM) algorithm to correct for recurrent mutation.
(0.08 MB DOC)

Text S4 Six-parameter model of chimpanzee evolution and estimation of parameters.
(0.08 MB DOC)

Text S5 Coalescent simulation of chimpanzee history.
(0.05 MB DOC)

Text S6 Simplified three-parameter model of chimpanzee evolution.
(0.05 MB DOC)

Text S7 Numerical procedure to identify migration models consistent with our data.
(0.05 MB DOC)

Text S8 CBHOM data and analysis of incomplete lineage sorting.
(0.06 MB DOC)

Text S9 Laboratory-based validation of 11 regions of incomplete lineage sorting.
(0.04 MB DOC)

Text S10 Filters used for preparing a clean data set for analysis.
(0.04 MB DOC)
