# Supplementary material for: Analysis of Chimpanzee History Based on Genome Sequence Alignments
Source: PLoS Genet. 2008 Apr 18;4(4):e1000057. doi: 10.1371/journal.pgen.1000057 (PMC2278377; doi:10.1371/journal.pgen.1000057)
Supplement: Table S1 — Stability of inferences using different data filters (0.06 MB DOC) [file pgen.1000057.s001.doc]

**Table S1: Stability of inferences using different data filters**

*Bold indicates the filter used in our main analysis; we chose the value that generated the largest number of divergent sites for analysis while producing stable results.*

***Divergent sites within a specified number of bases of other divergent sites***

|  | 5 | 4 | 3 | 2 | **1** | 0 |
| --- | --- | --- | --- | --- | --- | --- |
| total divergent sites | 476,672 | 538,604 | 609,833 | 695,011 | **802,121** | 1,007,985 |
| N_C_/N_W_ | 11.9 | 10.9 | 13.3 | 13.1 | **12.9** | 11.4 |
| t_ECW_/t_WW_ | 1.09 | 1.07 | 1.15 | 1.15 | **1.14** | 0.98 |
| t_ECWB_/t_WW_ | 2.91 | 2.98 | 2.84 | 2.87 | **2.89** | 2.73 |

Note: The values of several key statistics are stable if we filter out divergent sites within 1, 2, 3, 4, or 5 base pairs of another divergent site (t_WW_ refers to within-western chimpanzee heterozygosity). When we filtered out divergent sites within 1 or more bases of another divergent site we obtained stable results.

***Alignments with evidence for rate asymmetry***

|  | P<0.1 | P<0.01 | **P<0.001** | P<0.0001 | P<0.00001 | No filter |
| --- | --- | --- | --- | --- | --- | --- |
| total divergent sites | 773,660 | 801,156 | **802,121** | 802,239 | 802,309 | 802,597 |
| N_C_/N_W_ | 12.2 | 12.9 | **12.9** | 12.9 | 12.7 | 13.1 |
| t_ECW_/t_WW_ | 1.19 | 1.14 | **1.14** | 1.12 | 1.11 | 1.11 |
| t_ECWB_/t_WW_ | 2.99 | 2.89 | **2.89** | 2.90 | 2.90 | 2.89 |

Note: We calculated the probability that two branches of the tree would be as asymmetric as observed if the molecular clock holds (2-sided test). We obtained stable results when we filtered out regions that failed the symmetry test at a significance of P<0.001.

*Alignments showing high within-population diversity (suggesting duplicated regions)*

|  | π > 0.005 | π > 0.01 | **π > 0.02** | π > 0.03 | no filter |
| --- | --- | --- | --- | --- | --- |
| total divergent sites | 762,914 | 793,425 | **802,121** | 804,394 | 806,487 |
| N_C_/N_W_ | 17.1 | 13.3 | **12.9** | 12.4 | 11.4 |
| t_ECW_/t_WW_ | 1.33 | 1.24 | **1.14** | 1.08 | 0.98 |
| t_ECWB_/t_WW_ | 3.18 | 3 | **2.89** | 2.77 | 2.73 |

Note: We examined all pairs of reads from the same population but different individuals that mapped to a locus (usually pairs of western chimpanzee reads, since we had the largest number from this species). We filtered out all alignments where the divergence between two reads of the same population was greater than specified in the column header. Since this can filter out regions with deep genealogies, which would bias our analysis, we used a non-aggressive filter of π > 0.02, above which point we obtained stable results.
