# Supplementary material for: Analysis of Chimpanzee History Based on Genome Sequence Alignments
Source: PLoS Genet. 2008 Apr 18;4(4):e1000057. doi: 10.1371/journal.pgen.1000057 (PMC2278377; doi:10.1371/journal.pgen.1000057)
Supplement: Table S2 — Comparisons of genetic divergences (0.04 MB DOC) [file pgen.1000057.s002.doc]

**Text S2**

*Consistency of our data with Fischer et al.*

To check that our data set is consistent with previously reported data, we compared it to the published data set of Fischer et al[[1]](#footnote-2). The relevant portion of that data set for our study consists of 9 bonobos, 10 eastern, 10 central, and 10 western chimpanzees, sequenced over 26 regions. (We thank Anne Fischer for sharing this data in a format in which she provided the ancestral allele for all sites, determined by comparison with an outgroup.)

We calculated various statistics on these data, using a weighted jackknife analysis to obtain standard errors[[2]](#footnote-3),[[3]](#footnote-4), and then compared these statistics to our data and its jackknife error to assess whether the measurements were consistent (calculating a normally distributed Z-score).

**Text 2 Table 1: Comparison of statistics between our data and previously published data**

| **Statistic compared between the 2 data sets** | **4-species alignment used for our data** | **Our mean** | **Our stand-ard error** | **Fischer mean** | **Fischer stand-ard error** | **Z-score** | **Stand-ard error ratio** |
| --- | --- | --- | --- | --- | --- | --- | --- |
| WW_div_/WC_div_ | W_1_W_2_CH | 0.365 | 0.004 | 0.424 | 0.052 | -1.1 | 12.8 |
| WC_synap_/WW_synap_ | W_1_W_2_CH | 0.138 | 0.005 | 0.093 | 0.047 | 1.0 | 9.9 |
| CC_div_/CW_div_ | C_1_C_2_WH | 0.913 | 0.009 | 0.874 | 0.071 | 0.6 | 8.0 |
| CC_synap_/CW_synap_ | C_1_C_2_WH | 0.769 | 0.030 | 0.521 | 0.248 | 1.0 | 8.2 |
| WC_div_/(0.5(WB_div_+CB_div_)) | CWBM | 0.539 | 0.018 | 0.666 | 0.080 | -1.6 | 4.5 |
| (WB_synap_+CB_synap_)/WC_synap_ | CWBM | 0.200 | 0.026 | 0.409 | 0.155 | -1.3 | 5.9 |
| CB_synap_/WB_synap_ | CWBM | 0.833 | 0.197 | 0.628 | 0.611 | 0.3 | 3.1 |
| EC_div_/(0.5(EW_div_+CW_div_)) | ECWH | 0.925 | 0.022 | 0.907 | 0.074 | 0.2 | 3.4 |
| (EW_synap_+CW_synap_)/EC_synap_ | ECWH | 1.480 | 0.144 | 2.539 | 1.082 | -1.0 | 7.5 |
| CW_synap_/EW_synap_ | ECWH | 1.225 | 0.142 | 0.859 | 0.275 | 1.2 | 1.9 |
| EC_div_/CC_div_ | ECWH+C_1_C_2_WH | 1.003 | 0.031 | 1.001 | 0.047 | 0.0 | 1.5 |
| WW_div_/CC_div_ | W_1_W_2_CH+C_1_C_2_WH | 0.403 | 0.007 | 0.485 | 0.083 | -1.0 | 12.3 |
| F_ST_(EC) | EC-SNPs (n=103) | 0.154 | 0.031 | 0.136 | 0.027 | 0.4 | 0.9 |
| F_ST_(WC) | WC-SNPs (n=110) | 0.660 | 0.037 | 0.554 | 0.049 | 1.7 | 1.3 |
| F_ST_(WE) | EW-SNPs (n=134) | 0.690 | 0.032 | 0.593 | 0.048 | 1.7 | 1.5 |

Note: “div” indicates divergence per base pair between the two populations, or between two individuals from one population, while “synap” indicates clustering patterns. For example, WC_div_ is the difference per base pair between a western and central chimpanzee, while WC_synap_ in a W_1_W_2_CH alignment is the rate of divergent sites that cluster together a western and central chimpanzee to the exclusion of the other western chimpanzee and human. F_ST_ is calculated by comparing the Fischer et al. data to our genotyping of 304 SNPs discovered in an ECWHM alignment (Text S1). The letters in parentheses designate the populations being compared, and we restrict the calculation to a subset of SNPs that were discovered as divergent sites when considering chromosomes drawn from two different individuals. We calculate this in the Fischer et al. data by weighting SNPs according to their probability of being discovered in this way, and in our data by random sampling of SNPs. F_ST_ values were only calculated for SNPs in which the allele frequency averaged across the two populations was between 5% and 95%.

Text 2 Table 1 summarizes the comparisons of 15 statistics between the two data sets. We discuss a few quantities that are of interest:

• WW_div_/CC_div_ is the ratio of the per-base-pair divergence in two western chimpanzees, compared with that in two central chimpanzees. Our estimate is 0.403 ± 0.007, while for Fischer et al. it is 0.485 ± 0.083. These results are within error (Z = -1.0 standard deviation difference, which is non-significant assuming a normally distributed Z-score).

• WC_synap_/WW_synap_ is the ratio of the rates at which a western and central chimpanzee and at which two western chimpanzees cluster to the exclusion of all other populations in a W_1_W_2_CH alignment. The observed rate in our data is 0.138 ± 0.005, which is consistent with the 0.093 ± 0.047 from Fischer et al (Z = 1.0).

• F_ST_(EC) indicates the F_ST_ calculated between eastern and central chimpanzees by the Weir-Cockerham method[[4]](#footnote-5), using SNPs ascertained as divergent sites between a single eastern and single central chimpanzee sequence (we obtain this in the Fischer et al. data by weighting each SNP according to its probability of being discovered as this type of divergent site, and in our data from the subset of 103 SNPs from the 304 genotyped from our ECWHM alignment that could be considered to be discovered between only an eastern and central chimpanzee) (Text S1). The observed rate is 0.154 ± 0.031 in our data, which is consistent with the 0.136 ± 0.027 in Fischer et al (Z = 0.4).

Two features of these results are particularly notable. First, the Z-scores testing for a difference between Fischer’s estimates and ours are all non-significant (-1.6 < Z < 1.7 for the 15 statistics assessed). Thus, our estimates of parameters of chimpanzee population structure seem consistent with previous studies. Second, the standard errors from our analyses are 1.5 to 12.8 times less than Fischer et al., except for the F_ST_’s. This increased precision underlies our improved estimates of many parameters.

1. Fischer A, Pollack J, Thalmann O, Nickel B, Paabo S (2006) Demographic history and genetic differentiation in apes. Curr Biol. 16: 1133-1138. [↑](#footnote-ref-2)
2. Frank M, Meijer E, van der Leeden R (1999) Delete-m jackknife for unequal m. Statistics and Computing 9: 3-8. [↑](#footnote-ref-3)
3. Patterson N, Richter DJ, Gnerre S, Lander ES, Reich D (2006) Genetic evidence for complex speciation of humans and chimpanzees. Nature 441: 1103-1108. [↑](#footnote-ref-4)
4. Weir BS, Cockerham CC (1984) Estimating f-statistics for the analysis of population structure. Evolution 38: 1358–1370. [↑](#footnote-ref-5)
