# Supplementary material for: Analysis of Chimpanzee History Based on Genome Sequence Alignments
Source: PLoS Genet. 2008 Apr 18;4(4):e1000057. doi: 10.1371/journal.pgen.1000057 (PMC2278377; doi:10.1371/journal.pgen.1000057)
Supplement: Table S3 — “Rate tests” of the chimpanzee molecular clock (0.04 MB DOC) [file pgen.1000057.s003.doc]

**Text S3**

*Expectation Maximization (EM) algorithm to correct for recurrent mutation*

*Overview of algorithm*

To estimate the branchlengths of a genealogical tree averaged across the genome, the most straightforward approach is to count the relatives rates of the seven possible types of divergent sites, e.g., of C, W, B, H, CW, CB, and WB sites in a four-group CWBH alignment (Figure 1 in main text). Unfortunately, two or more mutations sometimes occur at the same site, and thus some divergent sites can in fact be due to two historical mutations. This is expected to inflate the estimated rates of rare divergent sites in alignments of four or more groups (e.g. CB and WB), as these are easily generated by recurrent mutation but are rare overall (Table S4). For inferences about population divergence times and size changes it is essential to correct for this error, as it can result in overestimation of short branches and thus of ancestral population sizes.

To correct for recurrent mutation, our main analyses were performed on data from five-group alignments (W_1_W_2_CHM, C_1_C_2_WHM, CWBHM, ECWHM), which are distinguished from the four-group alignments in that we add in a second outgroup species (a macaque). To analyze the data, we started with the same expectation maximization (EM) software that we recently described in a study of human-chimpanzee-gorilla alignments[[1]](#footnote-2).

The EM software takes advantage of the fact that in a five-group alignment, there are divergent site types that cannot have been generated due to a single historical mutation. For example in a CWBHM alignment, there are 15 different possible types of divergent sites, including 6 that we assume could not have been generated by a single historical mutation: CH, WH, BH, CWH, CBH, and WBH (Table S5). By studying the relative rates of these sites that are inconsistent with a single historical mutation, the EM algorithm estimates the proportion of each divergent site class that is due to recurrent mutation and estimates the true rate of each possible divergent site classError: Reference source not found.

The EM algorithm-based correction has a subtle but important effect on some analyses. Although the correction does not appreciably affect the estimates of pairwise genetic divergence for pairs of individuals—so that four- and three-group alignments produce excellent estimates of divergences between pairs of chimpanzee populations (Table 1 and Table S2)—the correction does affect the estimates of rates of unusual divergent site classes (Table S5). These sites are important for the inferences of demographic parameters (Tables 2-3 in the main text).

*Two new features of the EM algorithm specific to this study*

The EM algorithm we use makes the simplification that mutation rates (probability of a site mutating 0 times, 1 time, or 2 times) and the branchlengths of genealogical trees are constant across all loci. With these simplifications, we then use expectation maximization (EM) machinery[[2]](#footnote-3) to search for the combination of mutation rates and branchlengths, and the proportion of sites due to each number of mutations, that maximizes the likelihood of the observed counts of divergent site types in the data. The details of the computational implementation of the EM are described in ref. Error: Reference source not found; here, we describe two additions to the software that are specific for this study.

(1) We assumed the genealogical trees all are symmetric. This seems justified based on “rate tests” showing that mutation rates have remained unchanged since the split of humans and chimpanzees (ref. Error: Reference source not found and Table S3). Enforcing symmetry of the genealogical tree as a constraint in the EM algorithm has the benefit of reducing the number of branchlengths that need to be estimated from 8 to 4. For example in a CWBHM alignment, the 8 branchlengths are related by 4 equalities W=C, WB=CB, W+CW=B+CB, and W+CW+WB+CWB=H. As we had to estimate fewer parameters with the data after imposing the symmetry constraints, we were able to obtain more precise estimates of parameters. Encouragingly, we found that the qualitative results of Table 2 are unchanged compared with the same analyses when no symmetry is enforced.

(2) We applied a correction to the branchlength estimates to address a bias we discovered in the course of our simulations. This bias results from the (erroneous) assumption that genetic relationships are constant across the genome. This is a fairly reasonable approximation for the human-chimpanzee-gorilla genealogical trees, but for the more recently diverged chimpanzee populations, branchlengths vary dramatically relative to the genome-wide average. As the rate of recurrent mutations is proportional to the *square* of branchlengths, high variability can systematically affect estimates and a correction needs to be made based on this variability.

We quantified the magnitude of this bias by carrying out coalescent simulations in which we generated data under a reasonable model of history (Text S5), and then applied the EM analysis to estimate branchlengths. We found that while the branchlength estimates were close to the truth, they were also systematically biased relative to the truth. To understand the bias, we carried out simulations that matched the parameters of demographic history specified in Table 2, with three additional parameters values selected to produce branchlengths similar to what we observe in the real data. First, we simulated human-chimpanzee population separation to have occurred 250,000 generations ago with an ancestral population size of 50,000. Second, we simulated human-macaque population separation to have occurred 1,942,613 generations ago with an ancestral population size of 10,000. Third, we set the recurrent mutation parameter to match our data.

Text 3 Figure 1 shows the bias that emerges under these settings. In a CWBHM alignment, the WB and CB branchlengths that emerge from the EM analysis are inflated by a factor of 1.1084 compared with the true value used in simulation. More subtle biases include the fact that the short branchlengths (W and C) are inflated by 1.0046; the long branch-length (B) by 1.0084; and the human-macaque to human-chimpanzee divergence ratio by 1.0070. When we simulate data without recurrent mutations, no bias is observed. These results indicate that these biases arise from a combination of recurrent mutation and an EM analysis that imperfectly corrects for it.

**Text 3 Figure 1: Subtle biases in branchlength estimates by the EM analysis**

**Text 3 Figure 1:** Estimates of key branchlengths for the C_1_C_2_WHM, W_1_W_2_CHM, and CWBHM data sets, simulated using demographic parameters designed to provide a close match our data (we used the values specified in Table 2 and the text of this note). For each data set, we performed computer simulations where we knew the underlying parameters, and explored how different estimates from the EM algorithm compared to these true parameters (We explored this for different values of the recurrent mutation parameter on the x-axis.) We found a subtle but measureable bias in repeated simulation studies with large data sets. The most extreme bias is observed in the left-most panel, where we took the ratio of the rare synapomorphy branchlengths in a CWBHM alignment (WB and CB) to human-macaque divergence tHM in the same alignment. We found that the EM analysis overestimates this quantity by about 1.11 compared with the truth in repeated simulations, using a recurrent mutation parameter (x-axis) of 0.08 that is chosen to produce divergent site counts closely matching our real data. We also found more subtle biases in branchlength estimates (right three panels) for ratios of the short branchlengths (e.g. W+WB in a CWBHM alignment), long branchlengths (e.g. B+WB in a CWBHM alignment), and ratio of human-macaque to human-chimpanzee divergence (all these quantities were measureably biased, but always by <1%). To account for these biases, we adjusted all branchlength estimations that emerged from our EM analysis by the deflation factors in the Table below. These deflation factors are learned from the simulations and a recurrent mutation parameter setting of 0.08.

**Text 3 Table 1: Deflations applied to different EM-based estimates of branchlength**s

|  | **W_1_W_2_CHM** | **C_1_C_2_WHM** | **ECWHM** | **CWBHM** |
| --- | --- | --- | --- | --- |
| Synapomorphy rate | 1.0362 | 1.0346 | 1.0346 | 1.1084 |
| Short branch | 1.0026 | 1.0055 | 1.0055 | 1.0046 |
| Long branch | 1.0042 | 1.0046 | 1.0046 | 1.0084 |
| Human-macaque/human-chimp divergence | 1.0070 | 1.0068 | 1.0068 | 1.0070 |

In Text 3 Table 2, we show that when we apply the corrections, there is only a subtle change in inferences about demography compared with not applying correction at all. The largest changes are a ~3% changes in the estimate of t_ECWB_, an ~8% change in the estimate of N_ECWB_, and a ~2% change in the estimate of N_ECW_, encouraging us in the use of our inference procedure with or without this correction.

**Text 3 Table 2: Effect of corrections to EM analysis on demographic parameter estimates**

| **Name of parameter** | **No EM correction** | **EM correction (Table 2 value)** | **Change factor** |
| --- | --- | --- | --- |
| t_ECW_ | 510,020 | 510,220 | 1.00 |
| t_ECWB_ | 1,257,400 | 1,292,480 | 0.97 |
| N_C_ | 119,877 | 117,998 | 1.02 |
| N_W_ | 9,151 | 9,133 | 1.00 |
| N_ECW_ | 15,672 | 15,978 | 0.98 |
| N_ECWB_ | 22,546 | 20,852 | 1.08 |

*Verification that we obtain reasonable estimates of branchlengths using corrected EM algorithm*

To assess whether our analysis gives reasonable estimates of parameters, we used the values that emerged from the corrected analysis to carry out eight independent sets of computer simulations (each containing three data sets with 50,000 alignments each). We then applied the EM-corrected inference procedure to estimate the underlying parameters and asked how often the 90% credible intervals included the true value used in the simulation. The best estimates appear to be unbiased, although the 90% credible intervals may be slightly too tight (Text 3 Table 3). In 17% of simulations the true values of the parameters are outside the credible intervals, even though we expect that they will only be outside of the credible intervals in 10% of simulations. It is possible that this inflation may reflect our only performing a limited number of simulations.

**Text 3 Table 3: How often are the true values are outside the 95% credible intervals?**

| **Name of parameter** | **Fraction of 8 simulations for which the true parameter value is outside the 90% credible interval from our 6-parameter bootstrap analysis** |
| --- | --- |
| t_ECW_ | 25% |
| t_ECWB_ | 12.5% |
| N_C_ | 25% |
| N_W_ | 25% |
| N_ECW_ | 12.5% |
| N_ECWB_ | 0% |

1. Patterson N, Richter DJ, Gnerre S, Lander ES, Reich D (2006) Genetic evidence for complex speciation of humans and chimpanzees. Nature 441: 1103-8. [↑](#footnote-ref-2)
2. Baum LE, Petrie T, Soules G, Weiss N (1970) A maximization technique occurring in the statistical analysis of probabilistic functions of Markov Chains. Ann Math Stat 41: 164-171. [↑](#footnote-ref-3)
