# Supplementary material for: Analysis of Chimpanzee History Based on Genome Sequence Alignments
Source: PLoS Genet. 2008 Apr 18;4(4):e1000057. doi: 10.1371/journal.pgen.1000057 (PMC2278377; doi:10.1371/journal.pgen.1000057)
Supplement: Table S4 — A paucity of sites due to recurrent mutation (0.04 MB DOC) [file pgen.1000057.s004.doc]

**Table S4: A paucity of sites due to recurrent mutation**

| Data set | Total divergent sites | Event type | No. of event of this type observed | Estimated fraction explained by recurrent mutation |
| --- | --- | --- | --- | --- |
| C_1_C_2_WHM | 208,824 | C_1_W or C_2_W | 975 | 1.9% |
| W_1_W_2_CHM | 567,074 | W_1_C or W_2_C | 721 | 4.2% |
| CWBHM | 26,223 | CB or WB | 54 | 6.9% |
| ECWHM | 41,835 | EW or CW | 196 | 2.1% |

Notes: Application of the EM algorithm (Note S4) infers that for each of the five-sequence alignments, only a small fraction of divergent sites are likely due to recurrent mutation. Even for the rarest event classes (above) only 1.9%-6.9% of observed sites are estimated to be due to recurrent mutation.
