# Supplementary material for: Analysis of Chimpanzee History Based on Genome Sequence Alignments
Source: PLoS Genet. 2008 Apr 18;4(4):e1000057. doi: 10.1371/journal.pgen.1000057 (PMC2278377; doi:10.1371/journal.pgen.1000057)
Supplement: Table S5 — Counts of divergent sites from 5-sequence alignments (0.09 MB DOC) [file pgen.1000057.s005.doc]

**Table S5: Counts of divergent sites from 5-sequence alignments**

*Expected values and branchlengths are calculated from the EM algorithm*

**C_1_C_2_**WHM data set

| Divergent site type | Observed # | Expected # | Proportion of overall tree  (± 1 jackknife standard error) |
| --- | --- | --- | --- |
| C_1_ | 2,192 | 2,120 | 0.01035 (± 0.00015) |
| C_2_ | 2,113 | 2,120 | 0.01035 (± 0.00015) |
| W | 2,302 | 2,353 | 0.01145 (± 0.00017) |
| H | 17,290 | 17,221 | 0.08028 (± 0.00047) |
| M | 168,776 | 168,767 | 0.81520 (± 0.00103) |
| C_1_C_2_ | 740 | 736 | 0.00355 (± 0.00013) |
| C_1_C_2_W | 13,986 | 14,050 | 0.06392 (± 0.00047) |
| C_1_W | 492 | 503 | 0.00245 (± 0.00008) |
| C_2_W | 483 | 503 | 0.00245 (± 0.00008) |
| C_1_H | 51 | 35 | -- |
| C_2_H | 42 | 35 | -- |
| WH | 63 | 47 | -- |
| C_1_C_2_H | 108 | 120 | -- |
| C_1_WH | 85 | 107 | -- |
| C_2_WH | 101 | 107 | -- |

**W_1_W_2_**CHM data set

| Divergent site type | Observed # | Expected # | Proportion of overall tree  (± 1 jackknife standard error) |
| --- | --- | --- | --- |
| W_1_ | 2,506 | 2,502 | 0.00450 (± 0.00006) |
| W_2_ | 2,522 | 2,502 | 0.00450 (± 0.00006) |
| C | 7,539 | 7,517 | 0.01342 (± 0.00012) |
| H | 47,014 | 47,107 | 0.07993 (± 0.00030) |
| M | 460,580 | 460,559 | 0.82154 (± 0.00064) |
| W_1_W_2_ | 5,400 | 5,394 | 0.00960 (± 0.00011) |
| W_1_W_2_C | 39,485 | 39,425 | 0.06514 (± 0.00031) |
| W_1_C | 336 | 379 | 0.00068 (± 0.00003) |
| W_2_C | 385 | 379 | 0.00068 (± 0.00003) |
| W_1_H | 66 | 39 | -- |
| W_2_H | 74 | 39 | -- |
| CH | 393 | 391 | -- |
| W_1_W_2_H | 493 | 515 | -- |
| W_1_CH | 140 | 163 | -- |
| W_2_CH | 141 | 163 | -- |

CWBHM data set

| Divergent site type | Observed # | Expected # | Proportion of overall tree  (± 1 jackknife standard error) |
| --- | --- | --- | --- |
| C | 311 | 301 | 0.01164 (± 0.00048) |
| W | 304 | 301 | 0.01164 (± 0.00048) |
| B | 580 | 604 | 0.02332 (± 0.00076) |
| H | 2,179 | 2,129 | 0.07864 (± 0.00126) |
| M | 20,856 | 20,854 | 0.80672 (± 0.00289) |
| CW | 331 | 332 | 0.01272 (± 0.00072) |
| CWB | 1,484 | 1,521 | 0.05326 (± 0.00133) |
| CB | 16 | 29 | 0.00103 (± 0.00018) |
| WB | 38 | 29 | 0.00103 (± 0.00018) |
| CH | 3 | 4 | -- |
| WH | 8 | 4 | -- |
| BH | 35 | 27 | -- |
| CWH | 44 | 45 | -- |
| CBH | 16 | 21 | -- |
| WBH | 18 | 21 | -- |

ECWHM data set

| Divergent site type | Observed # | Expected # | Proportion of overall tree  (± 1 jackknife standard error) |
| --- | --- | --- | --- |
| E | 433 | 436 | 0.01052 (± 0.00031) |
| C | 428 | 436 | 0.01052 (± 0.00031) |
| W | 483 | 471 | 0.01136 (± 0.00038) |
| H | 3,470 | 3,486 | 0.08043 (± 0.00107) |
| M | 33,703 | 33,704 | 0.81497 (± 0.00230) |
| EC | 132 | 132 | 0.00313 (± 0.00032) |
| ECW | 2,881 | 2,867 | 0.06449 (± 0.00109) |
| EW | 86 | 97 | 0.00229 (± 0.00018) |
| CW | 110 | 97 | 0.00229 (± 0.00018) |
| EH | 13 | 8 | -- |
| CH | 4 | 8 | -- |
| WH | 8 | 11 | -- |
| ECH | 27 | 29 | -- |
| EWH | 28 | 27 | -- |
| CWH | 29 | 27 | -- |
