# Supplementary material for: Analysis of Chimpanzee History Based on Genome Sequence Alignments
Source: PLoS Genet. 2008 Apr 18;4(4):e1000057. doi: 10.1371/journal.pgen.1000057 (PMC2278377; doi:10.1371/journal.pgen.1000057)
Supplement: Table S6 — Sequencing reads available for analysis (prior to filtering) (0.04 MB DOC) [file pgen.1000057.s006.doc]

**Table S6: Sequencing reads available for analysis (prior to filtering)**

| **Latin name** | **Common name (individual)** | **Reads used in study** | **Sequencing center** |
| --- | --- | --- | --- |
| *Pan troglodytes troglodytes* | Central (Masuku) | 381,679 | Broad Institute |
|  | Central (Clara) | 312,286 | Broad Institute |
|  | Central (Noemie) | 499,150 | Broad Institute |
| *Pan troglodytes verus* | Western (Clint) | 19,720,347 | Broad Institute, Washington University |
|  | Western (Karlien) | 462,444 | Broad Institute |
|  | Western (Yvonne) | 450,137 | Broad Institute |
| *Pan troglodytes schweinfurthii* | Eastern (PR01008) | 36,083 | Broad Institute |
| *Pan paniscus* | Bonobo | 26,495 | Broad Institute |
| *Macaca mulatta* | Macaque | 13,810,571 | Baylor, Venter Institute, Washington University |
| *Homo sapiens* | Human | Build 34 sequence | Human genome project collaboration |

Note: This table describes data that was available to us in 2005. The 9 genome sequence alignments described in Text 10 are entirely based on these data, and do not include new macaque and western chimpanzee data collected since that time.
