# Supplementary material for: Analysis of Chimpanzee History Based on Genome Sequence Alignments
Source: PLoS Genet. 2008 Apr 18;4(4):e1000057. doi: 10.1371/journal.pgen.1000057 (PMC2278377; doi:10.1371/journal.pgen.1000057)
Supplement: Text S2 — Consistency of our data with Fischer et al. (0.07 MB DOC) [file pgen.1000057.s008.doc]

**Text S8**

*CBHOM data and analysis of incomplete lineage sorting*

*Preparation of the data set*

To prepare the CBHOM data, which consisted of aligned sequence from chimpanzee, bonobo, human, orangutan, and macaque, we used a different procedure from what we used for the nine other data sets we studied. Details of our procedure will be described in subsequent studies (S. Mallick, S. Gnerre, D. Reich in preparation). In what follows, we summarize salient points.

(a) *We used whole-genome assemblies rather than reads*

Instead of building alignments out of the raw sequencing reads from each species, we used whole-genome assemblies of chimpanzee, orangutan, and macaque. The assemblies were built by S. Gnerre using the Arachne assembler[[1]](#footnote-2), and then assisted against Build 36 of the human reference sequence (S. Gnerre in preparation). This does not ‘humanize’ an assembly; rather, the reference genome is used as an independent source of information to validate both read-read alignments (to improve read usage), and link integrity (to improve long-range connectivity). The sequencing coverage of each of the genome assemblies (in bases of quality score ≥20) is 7.3x for chimpanzee, 6.2x for orangutan, and 6.3x for macaque.

We also improved Arachne’s algorithms to generate consensus to better deal with the assembly of diploid genomes. For sites at which we observed allelic discrepancies (potential differences between the sequenced individual’s two chromosomes) we chose the allele that is observed in the majority of reads, and in the case of a tie, chose the allele with higher quality consensus sequence.

An advantage of using the genome assemblies rather than the individual reads is that it increases the reliability of the data. Except for bonobo, we have multiple coverage of most bases in the genome, resulting in high consensus sequence quality. Another advantage is that the large regions of contiguous sequence that emerge from a genome assembly provide more specificity when alignments are made across species.

(b) *We did not restrict ourselves to a single read for each chimpanzee and bonobo species*

In the nine main data sets in this study, we restricted analysis to a single read from each chimpanzee and bonobo population, to ensure that only a single haplotype was analyzed. As a result, the alignments were on average only a couple of hundred base pairs in length. By contrast, our goal in the CBHOM analysis was to maximize the number of aligned positions, and we therefore used amalgamations of reads (consensus sequence from assemblies) where possible. To increase the specificity of the bonobo data in the absence of an assembly, we required that all the sequences we analyzed came from paired-end reads that mapped uniquely in opposite orientations and with the correct spacing onto the human reference sequence.

(c) *Pairwise alignments followed by multiple sequence alignments*

We used Blastz[[2]](#footnote-3) to construct pairwise alignments between the human genome reference sequence (Build 36) and each of the other species: chimpanzee, bonobo, orangutan, and macaque. Gap parameters and substitution matrices for each alignment were chosen by linearly interpolating between parameters that have been published as appropriate for human-chimp Blastz alignments[[3]](#footnote-4) and the parameters appropriate for human-mouse comparisonsError: Reference source not found. The specific parameters we used depended on the genetic divergence of each species from human.

Once the pairwise alignments were generated, we searched for regions of the genome where all five species were represented, and where the alignment was unbroken by a deletion of more than 100 bp in any of these species. The sequences for these regions were masked using RepeatFinder[[4]](#footnote-5) to eliminate short tandem repeats and regions of low complexity. The Clustalw program[[5]](#footnote-6) was then used to construct multiple sequence alignments and to identify divergent sites (using default parameters). The alignments we built are available on our website at http://genepath.med.harvard.edu/~reich/Data%20Sets.htm.

After building the alignments, we applied filters to the data:

(1) We excluded aligned bases that did not have i) a minimum PHRED quality score of at least 30 in all sequences (species) that were compared and ii) a minimum PHRED quality scores of at least 20 for 5 bases on either side in all sequences. Analogous to the analysis in Table S1, applying more stringent cutoffs than this did not produce substantial differences in our main estimates (results not shown).

(2) We excluded divergent sites within 5 bases of the end of an alignment, or 10 bases from an insertion/deletion polymorphism. Analogous to the analysis in Table S1, we established that applying more stringent cutoffs did not substantially affect our estimates (results not shown).

(3) We excluded regions with less than 100 bases of sequence aligned from all groups.

(4) We excluded CpG divergent sites from analysis because they are known to mutate at a higher rate than other sites in the genome and thus are likely to be enriched in divergent sites that arose due to recurrent mutation

(5) We excluded divergent sites with more than two alleles across all five groups.

(6) We excluded regions where there was evidence of an unusually high accumulation of mutations on any one side of a genealogical tree. Essentially, we were looking for loci where there was an extreme molecular clock violation, which would suggest misalignment. To implement this, for 7 comparisons—bonobo-chimpanzee, bonobo-human, bonobo-orangutan, chimpanzee-human, chimpanzee-orangutan, human-orangutan, and orangutan-macaque—we tested whether the partitioning of divergent sites on either side of the tree (using the other species as outgroups where appropriate), was different from the genome-wide average. Any alignment that was significantly different compared with the average (P<0.001) was rejected.

(7) We excluded divergent sites from analysis if they were within 1 base pair of another site, following the rule from the other nine data sets (Text S10).

After application of these filters, summary statistics such as the ratio of chimpanzee-bonobo divergence to human-chimpanzee divergence were the same as in other data sets (Table 1).

*Identification of alignments that strongly favor BH or CH clustering*

We screened through the 18,985 CBHOM alignments that emerged from our filtering procedure, searching for ones where a genealogy clustering BH or CH was strongly favored.

We simplified the problem by comparing three alternative genealogies with fixed branch lengths, and testing for the best fit to the data. For the CB genealogy, we assumed the branchlengths were the same as the genome average. For the alternative genealogies (BH and CH), we assumed that the human-only, chimpanzee-only, and bonobo-only branches were about equal in length, and that the branch ancestral to the clustering species was about a third of that length. The relative probabilities of H:C:B:CH:BH:CB sites for the three models that we tested, which we normalized so that the probabilities of these six classes of sites summed to 1, are presented in Text 8 Table 1.

**Text 8 Table 1: Relative probabilities of divergent sites for the three genealogies**

Using the three models specified in this table, we tested each of the 18,985 alignments for the relative likelihoods of the data being observed for each of the three proposed genealogical trees. We then filtered the results to remove alignments that were a poor fit to any of the three models (P<0.01 comparing empirically to all alignments by a χ^2^ statistic), and where visual inspection indicated odd alignment properties. After these filters were applied there were 11 alignments that strongly support unusual genealogies, with >20,000:1 likelihood of CH or BH clustering over CB clustering (Table 4).

1. Jaffe DB, Butler J, Gnerre S, Mauceli E, Lindblad-Toh K, Mesirov JP, Zody MC, Lander ES (2003) Whole-genome sequence assembly for mammalian genomes: Arachne 2. Genome Research 13: 91-96. [↑](#footnote-ref-2)
2. Schwartz S, Kent JW, Smit A, Zhang Z, Baertsch R, Hardison R, Haussler D, Miller W. (2003) Human-Mouse Alignments with BLASTZ. Genome Research 13: 103-107. [↑](#footnote-ref-3)
3. Kvikstad EM, Tyekucheva S, Chiaromonte F, Makova KD (2007) A Macaque's-Eye View of Human Insertions and Deletions: Differences in Mechanisms. PLoS Comput Biol 3: e176 [↑](#footnote-ref-4)
4. Smit, AFA, Hubley, R & Green, P. *RepeatMasker Open-3.0*.1996-2004 <http://www.repeatmasker.org> [↑](#footnote-ref-5)
5. Higgins D, Thompson J, Gibson T, Thompson JD, Higgins DG, Gibson TJ. (1994) CLUSTAL W: improving the sensitivity of progressive multiple sequence alignment through sequence weighting,position-specific gap penalties and weight matrix choice. Nucleic Acids Research 22: 4673-4680. [↑](#footnote-ref-6)
