# Supplementary material for: Analysis of Chimpanzee History Based on Genome Sequence Alignments
Source: PLoS Genet. 2008 Apr 18;4(4):e1000057. doi: 10.1371/journal.pgen.1000057 (PMC2278377; doi:10.1371/journal.pgen.1000057)
Supplement: Text S4 — Six-parameter model of chimpanzee evolution and estimation of parameters. (0.08 MB DOC) [file pgen.1000057.s010.doc]

**Text S4**

*Six-parameter model of chimpanzee evolution and estimation of parameters*

The rates of divergent sites corrected for recurrent mutation can be used to infer parameters that model the evolutionary history of bonobos and chimpanzees. We assume an evolutionary model that allows for two separation times and four freely mixing, constant-size population sizes, a total of six parameters (t_ECW_, t_ECWB_, N_C_, N_W_, N_ECW_, N_ECWB_) (Figure 1b). We further assume a constant rate of divergent site accumulation throughout evolutionary history. This assumption of a “molecular clock” is reasonable, as shown by the “rate tests”[[1]](#footnote-2) in Table S3.

To identify values of these six parameters that are most consistent with the data, we use information from three five-group alignments: C_1_C_2_WHM, W_1_W_2_CHM and CWBHM. We ignore eastern chimpanzees in these analyses. We use an expectation maximization (EM) analysis (*Materials and Methods*) (Text S3) to estimate branchlengths for each data set correcting for recurrent mutations, using lengths normalized to the human-macaque divergence as inputs to the EM software. The seven values we estimated were:

***t_C_*** the average time since the last common ancestor of two central chimpanzee sequences (derived from C_1_C_2_WHM data)

***t_W_*** the average time since the last common ancestor of two western chimpanzee sequences (W_1_W_2_CHM data)

***t_P_*** the average time since the last common ancestor of a central and western sequence (weighted average of W_1_W_2_BHM, C_1_C_2_WHM and CWBHM data)

***t_B_*** the average time since the last common ancestor of a chimpanzee and bonobo sequence (CWBHM data)

$\text{t}_{\text{C}_{1}\text{W}}$ the rate at which a C_1_W (or C_2_W) divergent site occurs (C_1_C_2_WHM data)

$\text{t}_{\text{W}_{1}\text{C}}$ the rate at which a W_1_C (or W_2_C) divergent site occurs (W_1_W_2_CHM data)

***t_CB_*** the rate at which a CB (or WB) divergent site occurs (CWBHM data)

*Step 1: Inferring parameters associated with western-central population divergence*

To relate these values to the parameters of the model, we begin by considering the evolutionary history of central chimpanzees and western chimpanzees, which is defined by three of our parameters (t_ECW_, N_C_, N_W_), and two additional parameters that are associated with coalescence times in the ancestral population (K, R). For this part of the calculation, we consider only the C_1_C_2_WHM and W_1_W_2_CHM data sets (the largest five-sequence data sets we studied).

We define K as the expected time to the common ancestor for two central chimpanzee genes (or alternatively two western chimpanzee genes), assuming that they trace their lineages as far as central-eastern population separation t_ECW_ without sharing a common ancestor. In other words, K corresponds to the expected time since the common ancestor in the population just ancestral to western chimpanzee divergence. This would be the effective population size in that population if that population was constant in size. With this formulation, we can relate the average time since the last common ancestor of two central chimpanzee sequences to t_ECW_, N_C_, N_W_, and K as follows:

(1) ***t_C_*** $=$ P(coalescence at time < t_ECW_)(average coalescence time in this case) +

P(coalescence at time ≥ t_ECW_)(average coalescence time in this case)

= $\frac{1}{2N_{C}}\int_{0}^{t_{ECW}} te^{-t/2N_{C}}dt+e^{-t_{ECW}/2N_{C}}\left(t_{ECW}+K\right)$

= $2N{}_{C}+e^{-t_{ECW}/2N_{C}}\left(K-2N_{C}\right)$

The average time since the last common ancestor of two western chimpanzee sequences is:

(2) ***t_W_*** $=$ $2N_{W}+e^{-t_{ECW}/2N_{W}}\left(K-2N_{W}\right)$

The average time since the ancestor of a western and central chimpanzee is:

(3) ***t_P_*** = t_ECW_ + K

We now define R to be the expected time since the common ancestor of two lineages in the ancestral chimpanzee population, if this corresponds to the second coalescence of three lineages prior to the central chimpanzee-western chimpanzee speciation. The rate of a particular type of divergent site not consistent with the population tree (e.g. W_1_C in a W_1_W_2_CHM alignment) will be equal to one-third R, multiplied by the probability of coalescence not occurring at time < t_ECW_. We expect R = K if the population ancestral to western and central chimpanzees was constant in size. If N_ECW_ = N_ECWB_, K = R = 2N_ECW_ = 2N_ECWB_.

(4) $\text{t}_{\text{C}_{1}\text{W}}$ = $\frac{R}{3}e^{-t_{ECW}/2N_{C}}$

(5) $\text{t}_{\text{W}_{1}\text{C}}$ = $\frac{R}{3}e^{-t_{ECW}/2N_{W}}$

Solving for *R* in equations (4) and (5), we obtain

(6) 2N_W_ =$\frac{t_{ECW}}{\frac{t_{ECW}}{2N_{C}}+\ln\left(\frac{\text{t}_{\text{C}_{1}\text{W}}}{\text{t}_{\text{W}_{1}\text{C}}}\right)}$

Finally, we can use equation (3) to solve for K = ***t_P_*** - t_ECW_, and substitute this equality and the equality in (6) into equations (1) and (2) to obtain:

(7) ***t_C_*** =$2N_{C}+e^{-t_{ECW}/2N_{C}}\left(\text{t}_{\text{P}}-t_{ECW}-2N_{C}\right)$

(8) ***t_W_*** $=$ $\left(\frac{t_{ECW}}{\frac{t_{ECW}}{2N_{C}}+\ln\left(\frac{\text{t}_{\text{C}_{1}\text{W}}}{\text{t}_{\text{W}_{1}\text{C}}}\right)}\right)+\frac{\text{t}_{\text{W}_{1}\text{C}}}{\text{t}_{\text{C}_{1}\text{W}}}\times e^{-t_{ECW}/2N_{C}}\times\left(\text{t}_{\text{P}}-t_{ECW}-\frac{t_{ECW}}{\frac{t_{ECW}}{2N_{C}}+\ln\left(\frac{\text{t}_{\text{C}_{1}\text{W}}}{\text{t}_{\text{W}_{1}\text{C}}}\right)}\right)$

We can then solve numerically for t_ECW_, N_C_, N_W_, K, and R.

*Step 2: Inferring parameters associated with bonobo divergence*

To obtain estimates for the remaining three parameters of the model (t_ECWB_, N_ECW_, N_ECWB_), we use the estimate obtained for the parameter t_ECW_ from the analysis above and then use information from the smaller-size CWBHM data set. First, we expand equation (3) to a more detailed form that allows for changes in size of the ancestral populations:

(9) ***t_P_*** $=$ P(coalescence at t_ECW_ ≤ time < t_ECWB_)(average coalescence time in this case) + P(coalescence at time ≥ t_ECWB_)(average coalescence time in this case)

= $\frac{1}{2N_{ECW}}\int_{0}^{t_{ECWB}-t_{ECW}} \left(t_{ECW}+t\right)e^{-t/2N_{ECW}}dt+\frac{1}{2N_{ECWB}}e^{-\left(t_{ECWB}-t_{ECW}\right)/2N_{ECW}}\int_{0}^{\infty} \left(t_{ECWB}+t\right)e^{-t/2N_{ECWB}}dt$

$$=t_{ECW}+2N_{ECW}+e^{-(t_{ECWB}-t_{ECW})/2N_{ECW}}(2N_{ECWB}-2N_{ECW})$$

The average time since the last common ancestor of a chimpanzee and a bonobo is:

(10) ***t_B_*** = t_ECWB_ + 2N_ECWB_

The rate of a type of divergent site not consistent with the population tree is:

(11) ***t_CB_*** $=$ $\frac{2\text{N}_{\text{ECWB}}}{3}e^{-\left(\text{t}_{\text{ECWB}}-\text{t}_{\text{ECW}}\right)/2\text{N}_{\text{ECW}}}$

We use equation (10) to solve for t_ECWB_ = ***t_B_*** – 2N_ECWB_, substitute this equality into equation (11), and solve for 2N_ECW_:

(12) 2N_ECW_ =$\frac{t_{ECW}+2N_{ECWB}-\text{t}_{\text{B}}}{\ln\left(\frac{3\text{t}_{\text{CB}}}{2N_{ECWB}}\right)}$

We then substitute t_ECWB_ = ***t_B_*** – 2N_ECWB_, as before, and equation (12) into equation (9) to obtain:

(13) ***t_P_*** = $t_{E CW}+\frac{t_{ECW}+2N_{ECWB}-\text{t}_{\text{B}}}{\ln\left(\frac{3\text{t}_{\text{CB}}}{2N_{ECWB}}\right)}+\frac{3\text{t}_{\text{CB}}}{2N_{ECWB}}\times\left(2N_{ECWB}-\frac{t_{ECW}+2N_{ECWB}-\text{t}_{\text{B}}}{\ln\left(\frac{3\text{t}_{\text{CB}}}{2N_{ECWB}}\right)}\right)$

Now we can numerically solve for N_ECWB_, N_ECW_, and t_ECWB_.

*Computational implementation and data checking*

To implement this procedure computationally, we wrote a software program in PERL that obtains 90% credible intervals for parameters of interest using bootstrap analysis. Since each of the three data sets (W_1_W_2_CHM, C_1_C_2_WHM, and CWBHM) were divided into unlinked (and thus statistically independent) clusters, we were able to randomly resample these clusters (with replacement), and for each resampling, repeat the calculation above and determine the parameters of interest. The 90% credible interval for each parameter was obtained as the range between the 5^th^ and 95^th^ percentiles, based on 1,000 bootstrap resamplings of all three data sets together.

To assess the reliability of this inference procedure (and of the bootstrap credible intervals), we wrote a coalescent computer simulation (Text S5) to simulate data under our model. We used this simulation software to generate W_1_W_2_CHM, C_1_C_2_WHM, and CWBHM data sets, and applied the procedure described in this supplementary note to these simulated data to estimate the values of the underlying parameters of demographic history. Our analytical procedure generated accurate estimates of the parameters used to simulate data (at least for the scenarios we explored), even in the presence of data sets that harbor recurrent mutation (Text S5).

1. Sarich VM (1983) In: Ciochon RL, Corruccini RS, editors. New Interpretations of Ape and Human Ancestry. New York: Plenum. pp.137–150 [↑](#footnote-ref-2)
