# Supplementary material for: Analysis of Chimpanzee History Based on Genome Sequence Alignments
Source: PLoS Genet. 2008 Apr 18;4(4):e1000057. doi: 10.1371/journal.pgen.1000057 (PMC2278377; doi:10.1371/journal.pgen.1000057)
Supplement: Text S5 — Coalescent simulation of chimpanzee history. (0.05 MB DOC) [file pgen.1000057.s011.doc]

**Text S5**

*Coalescent simulation of chimpanzee history*

*Basic description of simulation*

We wrote a coalescent computer simulation based on an algorithm of Richard Hudson [[[1]](#footnote-2),[[2]](#footnote-3)]. The simulation generates data sets under the six-parameter model of chimpanzee history described in Text S4, with the addition of extra parameters:

• We added two parameters to allow us to simulate eastern chimpanzee data, under the assumption that they split suddenly from the central population. The additional parameters are t_EC_, the splitting time of eastern and central chimpanzees and N_E_, the modern eastern chimpanzee size.

• We added a parameter corresponding to the bonobo modern effective population size, N_B_.

• We added two parameters to allow migration between western and central chimpanzees in an “isolation and migration model”[[3]](#footnote-4). In this model, after the initial split of western and central chimpanzees, the two populations continued to exchange genes at a constant rate, with m_C_ specifying the proportion of central genes replaced by western genes per generation, and m_W_ specifying the proportion of western genes replaced by central genes per generation.

• We added a parameter giving the probability that at a site where a mutation had already occurred, a second mutation would also occur in the genealogy we were simulating. This allowed us to explore the effect of recurrent mutation on our data and analysis.

• We allowed an option to simulate large numbers of samples from each chimpanzee population, allowing us to simulate data sets similar to Fischer et al.[[4]](#footnote-5), or similar to the data we ourselves generated by genotyping SNPs discovered in alignments of five shotgun sequences.

*Characteristics of simulation output*

Our simulation generated sequence alignments averaging 1,000 base pairs in length, designed to be similar to what we obtained after concatenating sequences for bootstrap and jackknife analysis. For each run of the simulation, we obtained four output files, corresponding to the W_1_W_2_CHM, C_1_C_2_WHM, CWBHM, and ECWHM alignments, in the same format as our real data. By running the simulation four times with different random number seeds and keeping a different alignment for each run, we obtained simulated data that is comparable to our real data.

We note that the simulations we used for this study do not include recombination. While this is unrealistic, it does not bias any of our results, since all of our inferences are based on average genetic divergence, which is not affected by recombination.

*Testing shows that the simulation meets theoretical expectations*

To test the reliability of the simulation, we checked that various statistics that could be measured on simulated data (such as rates of W, C, and WC divergent sites in a W_1_W_2_CHM alignment) met expectations from coalescent theoryError: Reference source not found.

To check that the migration parameters were working correctly, we used the fact that Donnelly and Tavare[[5]](#footnote-6) give an analytic result for a two-island model with equal migration (m_C_=m_W_) and equal sized populations (N_W_=N_C_). For this scenario, we expect coalescent times of alleles in the two populations to be 2N_C_ + 1/4m_C_, and we obtained this result from our simulations.

*We can recover the parameters used in computer simulation*

A strong test of whether the simulation is working properly—and also if the six-parameter inference procedure is working—is whether we can recover the parameters that were used, by applying the six-parameter inference procedure to the simulated data.

To implement this test, we simulated data under a set of parameters (Text 5 Table 1). We ran the simulation without recurrent mutations, using very large simulated data sets so we could use raw counts of divergent sites to estimate branchlengths: thus, the present analysis is not complicated by application of the EM algorithm.

**Text 5 Table 1: Test of simulations and our six**-parameter inference procedure shows that we can recover the parameters used in simulation

| **Name of parameter** | **Value of para-meter used in simulation** | **5^th^ percentile of bootstrap analysis** | **95th percentile of bootstrap analysis** |
| --- | --- | --- | --- |
| t_ECW_ | 25,501 | 24,032 | 26,515 |
| t_ECWB_ | 62,871 | 61,891 | 63,781 |
| N_C_ | 119,880 | 116,327 | 137,258 |
| N_W_ | 9,151 | 8,766 | 9,343 |
| N_ECW_ | 15,672 | 14,780 | 16,809 |
| N_ECWB_ | 22,546 | 22,213 | 23,281 |

Note: Times are given in generations, compared with years elsewhere in this study. We used very large simulated data sets to allow us to stringently test our parameter estimation.

As shown in Text 5 Table 1, the 90% credible intervals (from our six-parameter estimation with bootstrap analysis) include the true parameter used in the simulation. This suggests that both the simulation and six-parameter inference procedure (Text S4) are working as expected.

1. Hudson RR (1990) Gene genealogies and the coalescent process. Oxf Surv Evol Biol 7: 1–44. [↑](#footnote-ref-2)
2. Reich DE, Feldman MW, Goldstein DB (1999) Statistical properties of two tests that use multilocus data sets to detect population expansions. Mol Biol Evol 16: 453-466. [↑](#footnote-ref-3)
3. Nielsen R, Wakeley J (2001) Distinguishing migration from isolation: a Markov chain Monte Carlo approach. Genetics 158: 885–896. [↑](#footnote-ref-4)
4. Fischer A, Pollack J, Thalmann O, Nickel B, Pääbo S (2006) Demographic history and genetic differentiation in apes. Curr Biol 16: 1133-1138. [↑](#footnote-ref-5)
5. Donnelly and Tavare (1995) Coalescents and genealogical structure under neutrality. Annual Reviews of Genetics 29: 401-201 [↑](#footnote-ref-6)
