# Supplementary material for: Analysis of Chimpanzee History Based on Genome Sequence Alignments
Source: PLoS Genet. 2008 Apr 18;4(4):e1000057. doi: 10.1371/journal.pgen.1000057 (PMC2278377; doi:10.1371/journal.pgen.1000057)
Supplement: Text S6 — Simplified three-parameter model of chimpanzee evolution. (0.05 MB DOC) [file pgen.1000057.s012.doc]

**Text S6**

*Simplified three-parameter model of chimpanzee evolution*

In our main six-parameter model of chimpanzee evolution, we separately estimated N_ECW_ and N_ECWB_. As a result, we had to analyze the C_1_C_2_WHM, W_1_W_2_CHM, and CWBHM data sets jointly to estimate the parameters of the model. However, as a test of robustness we also repeated our analysis assuming that N_ECW_=N_ECWB_, which seems reasonable as we could not reject the hypothesis that they were equal: N_ECW_/N_ECWB_ = 0.77 (0.51-1.18) (Table 3).

Under this simplification, each data set can be separately used to infer parameters. In other words, C_1_C_2_WHM is sufficient to infer t_ECW_, N_C_ and N_ECW_=N_ECWB_, and W_1_W_2_CHM is sufficient to infer t_ECW_, N_W_ and N_ECW_=N_ECWB_. To obtain estimates of our key parameters under the simplified three-parameter model, we then compute a weighted average of the parameter estimates across the three data sets. Details of the three-parameter model for C_1_C_2_WHM are presented in what follows, where N_a_=N_ECW_=N_ECWB_:

(1) ***t_C_*** $=$ P(coalescence at time < t_1_)(average coalescence time in this case) + P(coalescence at time ≥ t_1_)(average coalescence time in this case)

= $\frac{1}{2N_{C}}\int_{0}^{t_{ECW}} te^{-t/2N_{C}}dt+e^{-t_{ECW}/2N_{C}}\left(t_{ECW}+2N_{a}\right)$

= $2N{}_{C}+e^{-t_{ECW}/2N_{C}}\left(2N_{a}-2N_{C}\right)$

(2) ***t_P_*** = t_ECW_ + 2N_a_

(3) $\text{t}_{\text{C}_{1}\text{W}}$ = $\frac{2N_{a}}{3}e^{-t_{ECW}/2N_{C}}$

Some algebra, similar to that described for the second half of the six-parameter model (Text S4), gives the following set of equations, which we solve numerically:

(4) ***t_C_*** = $\frac{2N_{a}-\text{t}_{\text{P}}}{\ln\left(\frac{3\text{t}_{\text{C}_{1}\text{W}}}{2N_{a}}\right)}+\frac{3\text{t}_{\text{C}_{1}\text{W}}}{2N_{a}}\times\left(2N_{a}-\frac{2N_{a}-\text{t}_{\text{P}}}{\ln\left(\frac{3\text{t}_{\text{C}_{1}\text{W}}}{2N_{a}}\right)}\right)$

(5) 2N_C_ = $\frac{2N_{a}-\text{t}_{\text{P}}}{\ln\left(\frac{3\text{t}_{\text{C}_{1}\text{W}}}{2N_{a}}\right)}$

(6) t_ECW_ = ***t_P_*** – 2N_a_

Similar logic applies to the W_1_W_2_CHM data set, where ***t_W_*** replaces ***t_C_***, $\text{t}_{\text{W}_{1}\text{C}}$ replaces $\text{t}_{\text{C}_{1}\text{W}}$, and N_W_ replaces N_C_.
