# Supplementary material for: Analysis of Chimpanzee History Based on Genome Sequence Alignments
Source: PLoS Genet. 2008 Apr 18;4(4):e1000057. doi: 10.1371/journal.pgen.1000057 (PMC2278377; doi:10.1371/journal.pgen.1000057)
Supplement: Text S7 — Numerical procedure to identify migration models consistent with our data. (0.05 MB DOC) [file pgen.1000057.s013.doc]

**Text S7**

*Numerical procedure to identify migration models consistent with our data*

The inference procedure in our paper—where we find a six-parameter model of central-western-bonobo history that can fit our data—does not work in the presence of migration, since we are not able to solve the equations analytically.

To address this, we implemented a computer program that obtains the expected branchlengths of the different event classes in the W_1_W_2_CHM, C_1_C_2_WHM, and CWBHM alignments from specified values of the six parameters in the main model, as well as specified values of western-to-central migration m_C_ and central-to-western migration m_W_. The program works in the opposite direction from our inference procedure – estimating branchlengths from parameters instead of parameters from branchlengths – enabling us to compare different combinations of parameters in terms of how well they correspond to the known branchlengths.

The program begins with eight input parameters: t_ECW_, t_ECWB_, N_C_, N_W_, N_WC_, N_BWC_, m_W_, and m_C_. The program then moves backward in time in fixed increments (100 generations per increment by default, although we explored smaller increments and found they did not produce substantially different results). For each time increment, the program then determines the probability that the ancestors of the three samples that are studied will be in a particular state (indexed by the population in which the ancestors live, and whether the samples have coalesced to a common ancestor). For example, in the CWBHM alignment:

In the interval 0 < t < t_ECW_, the samples can be in one of the following six states:

W ancestor in western population, C ancestor in central population

W ancestor in western population, C ancestor in western population

W ancestor in central population, C ancestor in central population

W ancestor in central population, C ancestor in western population

W and C ancestors coalesced and in western population

W and C ancestors coalesced and in central population

In the interval t_ECW_<t<t_ECWB_, the samples can be in one of the following two states:

W ancestor and C ancestor uncoalesced

W ancestor and C ancestor coalesced

In the interval t>t_ECWB_, the samples can be in one of the following five states:

W and C and B uncoalesced

WC coalesced, B uncoalesced

WB coalesced, C uncoalesced

CB coalesced, W uncoalesced

WCB all coalesced

In each of the three time intervals, we can calculate the transition probabilities among states. Going back in time in increments, multiplying the state vector by the transition probability matrix at each increment, we track the probability that the ancestors of the individuals today will be in each of the possible states, which in turn determines the branchlengths. We continue moving back in time until there is a 99.999% probability that all lineages are coalesced, to obtain the expected branchlengths for that demography.

We used the software to calculate expected branchlengths for the three different alignments W_1_W_2_CHM, C_1_C_2_WHM, and CWBHM under a specified demography. We then calculated the corresponding branchlengths in the real data, and summarized both the expected and observed data by seven summary statistics:

**Text 7 Table 1: Statistics for summarizing the W_1_W_2_CHM, C_1_C_2_WHM and CWBH**M data

| Statistic | Description |
| --- | --- |
| hetWC | western-central divergence |
| hetWB | western-bonobo divergence |
| synCWB | bonobo-western synapomorphy rate in a CWB alignment |
| hetWW | western-western divergence |
| synWWC | western-central synapomorphy rate in a WWC alignment |
| hetCC | central-central divergence |
| synCCW | western-central synapomorphy rate in a CCW alignment |

These parameters were estimated from our three real data sets (after correcting for multiple mutations by the EM algorithm), and then compared with the expectation from the numerical model by summing the quantity (observed-expected)×(observed-expected)/expected over all seven quantities, a figure-of-merit inspired by the chi-square statistic.

We used a hill-climb method to obtain a best fit of our model parameters to the real data. We start by fixing a value of the migration rate parameters. We then find the combination of the population sizes and split times that produce the best fit to the data. We start the hill-climb with a guess about the parameters (in practice we use the set of parameters that we obtain as our best estimate in the absence of migration; that is, the values given in Table 2). We then check whether the fit can be improved by multiplying or decreasing each of the parameters by a factor *L*. When we can not climb any higher, we reduce L and try again. The hill-climb stops when L < 1.005. The results, for different choices of the migration parameter in the symmetrical model, are shown in Figure 2 of the main paper.

As a test of whether the numerical procedure works, we simulated data under a set of demographic parameters (not allowing any migration between western and central chimpanzees), and then assessed how well the analysis reconstructed these parameters under the assumption of no migration. The match is excellent, as shown in Table N7b below.

**Text 7 Table 2**: Demonstration that the procedure can extract the parameters used in simulation

|  | m_C_=m_W_ | t_ECW_ | t_ECWB_ | N_W_ | N_C_ | N_WC_ | N_WCB_ |
| --- | --- | --- | --- | --- | --- | --- | --- |
| Parameters used in simulation | 0 | 28,266 | 64,169 | 9,552 | 123,806 | 13,885 | 20,752 |
| Parameters emerging from estimation | 0 | 28150 | 64,180 | 9,500 | 123,000 | 14,000 | 20,747 |
