# Supplementary material for: Analysis of Chimpanzee History Based on Genome Sequence Alignments
Source: PLoS Genet. 2008 Apr 18;4(4):e1000057. doi: 10.1371/journal.pgen.1000057 (PMC2278377; doi:10.1371/journal.pgen.1000057)
Supplement: Text S9 — Laboratory-based validation of 11 regions of incomplete lineage sorting. (0.04 MB DOC) [file pgen.1000057.s015.doc]

**Text S9**

*Laboratory-based validation of 11 regions of incomplete lineage sorting*

In Text S8 we identified 11 regions of the genome with strong evidence (>20,000:1 likelihood ratio) of incomplete lineage sorting comparing chimpanzees, bonobos and humans. In these regions, humans and chimpanzees cluster to the exclusion of bonobos (6 CH regions), or humans and bonobos cluster to the exclusion of chimpanzees (5 BH regions) (Table 4). Genetic divergence between bonobos and chimpanzees is also several times the genome-wide average, as expected if genetic divergence occurred prior to human-chimpanzee speciation.

We were concerned that these regions, which were drawn from the extreme tail of a distribution of 18,985 alignments, might reflect artifacts of our alignment procedure or other errors, rather than genuine regions of incomplete lineage sorting. To address this, we carried out PCR-based resequencing to validate and further study these regions. We targeted up to 5 kb centered on the 11 alignments identified in Text S8. We obtained consensus sequence for these regions by aligning the human and chimpanzee genome sequences (and orangutan where possible) obtained as in Text S8, and masking out sites where the species differed. We designed primers using the Primer3 software[[1]](#footnote-2) to cover as large a fraction of these regions as possible, in overlapping amplicons of up to 500 bases each.

Bidirectional sequencing was carried out using the ABI 3730 technology at the Broad Institute Sequencing and Analysis Platform. We sequenced DNA from 16 primates obtained from the Coriell Cell Repositories and the Max Planck Institute (Leipzig) as well as our own laboratory: 4 western chimpanzees (NS03629, NS06006 (Clint), Pearl, NA03450), 3 bonobos (PR00092, PR00251 and NG05253), 3 humans, 1 gorilla (NG05251), 2 orangutans (NA04272 and NA05252), and 3 macaques (NA03443, NA3446 and NG07101). A total of 35,351 bases passed quality control. The average number of bases successfully sequenced was lower in macaques (42% lower than the other species on average), presumably because differences between macaques and the other species interrupted primer sites. To maximize the sites we could analyze, we excluded the macaques from population genetic analyses.

Divergent sites among the species were identified by the SNP Compare software, which is an in-house software package developed at the Broad Institute that combines results from the PolyPhred version 5.0 software[[2]](#footnote-3) and the PolyDhan software (Richter et al. unpublished results). To check the reliability of the divergent site calls, we carried out two manual validation analyses.

(1) We inspected the sequencing traces at all 197 sites where the CBHOM shotgun sequence alignments overlapped sites identified from our resequencing data. There were no clear discrepancies between genotypes from shotgun sequencing data and the validation data.

(2) We inspected the sequence traces for 100 divergent sites that we chose for spot-checking and that did not overlap the sites from the original CBHOM analysis. We chose these sites by oversampling from the interesting classes. Specifically, we checked 13 chimpanzee-only sites, 16 bonobo-only sites, 13 human-only sites, 13 chimpanzee-bonobo clusters, 18 chimpanzee-human clusters, 18 bonobo-human clusters, and 9 chimpanzee-bonobo-human clusters. There was perfect agreement between the automated calls and our manual inspection at genotypes that were identified as homozygous by SNP Compare, encouraging us in the use of these data as the great majority of genotypes in our analyses were homozygous.

We next applied filters to eliminate sites where there was evidence of more than one mutation having occurred at the site. We eliminated:

(1) Sites where we observed more than two alleles

(2) Polymorphisms in hypermutable CpG dinucleotides (CG/TG or CG/CA polymorphisms)

(3) Polymorphisms shared across species (treating chimpanzees and bonobos as a single species)

(4) Polymorphisms shared across both the African apes (chimpanzees, bonobos, humans, and gorillas) and non-African primates (orangutans and macaques), suggesting multiple mutations.

We finally removed sites that overlapped the ones discovered in the CBHOM analysis, to ensure independence of the initial discovery of these regions from the data we collected to validate them.

After application of these filters, we obtained 1,408 sites that were polymorphic comparing chimpanzees, bonobos, humans, and orangutans (Table 5). There were 7 classes of sites that emerged from this alignment: chimpanzee-only (C), bonobo-only (B), human-only (H), chimpanzee-bonobo cluster (CB), chimpanzee-human cluster (CH), bonobo-human cluster (BH), and orangutan-only (O) (Table 5). Most of the sites fell into only one category, but if there was polymorphism within a species, all 7 classes were assigned weights according to their probability of being observed if a single chromosome was drawn from each species. Each divergent site had a maximum weight of 1, and the total sum of weights across all sites and classes was 1,264.

Table 5 presents an analysis of these data that confirms the evidence of incomplete lineage sorting. In 11 out of 11 regions, the divergent site class that was in excess in the shotgun analysis (e.g. CH in the 6 CH clusters, and BH in the 5 BH clusters), continues to be in excess compared with the opposite divergent site class (P<0.0005 by a binomial test). The genetic divergence between chimpanzees and bonobos averaged over these 11 regions (337) is 38.4% of that between humans and orangutans (878), compared with the 12.2% average genome-wide (Table 5). This ~3-fold excess divergence is expected if chimpanzees and bonobos shared a common ancestor prior to human-chimpanzee divergence in these regions

As a second line of analysis, Figure 5 presents the genetic patterns at increasing distances from the CBHOM alignments. As expected for a genealogy that spans only a limited distance in the genome, the estimated time since chimpanzee and bonobo shared a common ancestor decreases with increasing distance from the regions identified from genome sequence alignment.

1. Rozen S, Skaletsky H (2000) Primer3 on the WWW for general users and for biologist programmers. In: Bioinformatics Methods and Protocols: Methods in Molecular Biology, Krawetz S, Misener S, eds. Totowa, NJ, USA: Humana Press. 365–386. [↑](#footnote-ref-2)
2. Stephens M, Sloan JS, Robertson PD, Scheet P, Nickerson DA (2006) Automating sequence-based detection and genotyping of SNPs from diploid samples. *Nature Genetics* 38: 375-281. [↑](#footnote-ref-3)
