# Supplementary material for: Analysis of Chimpanzee History Based on Genome Sequence Alignments
Source: PLoS Genet. 2008 Apr 18;4(4):e1000057. doi: 10.1371/journal.pgen.1000057 (PMC2278377; doi:10.1371/journal.pgen.1000057)
Supplement: Text S10 — Filters used for preparing a clean data set for analysis. (0.04 MB DOC) [file pgen.1000057.s016.doc]

**Text S10**

*Filters used for preparing a clean data set for analysis*

We applied 10 filters to the 9 main genome sequence alignment data sets in this study. These filters enabled us to eliminate regions of potentially erroneous alignment, as well as sites that had an increased change of being in error. These restrictions are similar to those imposed in a related study in which we studied alignments of human, chimpanzee, gorilla, orangutan and macaque DNA[[1]](#footnote-2). In the descriptions of each successively applied filter below, we note differences with that study where they appear.

(1) We excluded bases that did not have a minimum quality score of at least 30 or five flanking bases on each side with minimum quality scores of at least 25.

(2) We excluded bases if they were inside, or within two bases of, a low-complexity region, as identified by the program Tandem Repeats Finder[[2]](#footnote-3). Regions of low-complexity are inappropriate for the analyses performed in this study for two reasons: they can represent highly mutable areas of the genome (microsatellites), and they are prone to misalignment.

(3) We excluded regions with less than 100 bases of sequence aligned from all groups; bases excluded according to the first two restrictions were not counted in this sum.

(4) We excluded regions where one of the groups had greater than 10 bases screened multiple times and exhibited an unusually high rate of heterozygosity (an extreme heterozygosity value often reflects the misalignment of at least one of the sequences). In the case of macaque heterozygosity, we excluded regions where macaque exhibited heterozygosity greater than three standard deviations above that expected from a Poisson distribution, assuming a heterozygosity rate of 0.002 differences per base.

We were more cautious in excluding regions where the chimpanzee populations exhibit high heterozygosity. Eliminating regions where chimpanzees exhibited high heterozygosity, if real, would selectively eliminate gene trees that coalesce in the ancestral population, leading us to underestimate both the rates of atypical clustering events and the variation in genetic divergence between chimpanzee groups throughout the genome.

We took the following steps to ensure that we excluded mostly poorly aligned regions based on rates of within-chimpanzee heterozygosity. In the alignments of two individuals from one population, the human-chimpanzee divergence measured in the individual that is truly highly heterozygous should be, on average, equal to the human-chimpanzee divergence measured in the other individual. If one individual from a population has two different haplotypes at a locus, the other individual, from the same population, should sometimes have one of these haplotypes and sometimes the other. However, if the high heterozygosity results from misalignment, the divergence between human and the highly heterozygous chimpanzee individual should be greater than that between human and the other chimpanzee individual.

We found that in regions where the within-individual heterozygosity was greater than 2 percent, the divergence between human and the heterozygous chimpanzee was greater than the average human-chimpanzee divergence in the region (data not shown). In regions where one chimpanzee group exhibited a heterozygosity value below 2 percent, there was a moderate elevation of human-heterozygous chimpanzee divergence that suggested a background rate of poorly aligned regions; however, when the chimpanzee has greater than 2% heterozygosity, this pattern is observed more frequently . We excluded this last set of regions from the analysis.

(5) We excluded regions where an unusually high number of reads from one group mapped to the same locus; such regions might reflect the erroneous alignment of segmental duplications. Specifically, we excluded regions if the number of reads from one of the groups divided by the length of the region in bases was greater than 0.02, as we empirically observed such regions to be outliers.

(6) We excluded regions where there was evidence of a significantly high accumulation of mutations on one part of the tree compared to the others, which was consistent with the effects of sequence misalignments or segmental duplications. To find such regions, we measured violations of the expectation of symmetry when considering separately each set of three groups’ aligned sequences. We calculated a binomial statistic measuring the deviation of the region from each of these expected symmetries, and excluded regions that violated any of the symmetry expectations with a p-value below 0.001. The significance level used in this study (α = 0.001) was more stringent than that used previously (α = 0.0001)Error: Reference source not found. We chose this new cutoff because we found that the rate of western chimpanzee individual-central chimpanzee clustering reached a stable value only after we increased the stringency to α = 0.001, indicating that one or more abnormal regions contributed heavily to the estimate at lower stringency levels.

(7) We excluded regions that mapped to known segmental duplications in humans or chimpanzees[[3]](#footnote-4).

(8) We excluded divergent sites from analysis if they were next to other divergent sites, as such crowding of events can reflect misaligned sequences. We chose to exclude only divergent sites that were within one base of another divergent site, because this restriction allowed our estimate of the divergence of the two most closely related groups to reach stable values.

Excluding divergent sites that are adjacent to other divergent sites might to some extent preferentially exclude information from regions with large gene tree depths. However, the restriction should not substantially affect our measures of the variability in chimpanzee branchlengths, as this variability contributes only slightly to the variability in overall tree branchlength. For example, excluding sites next to divergent sites does not substantially change the estimated rates of atypical clustering events, even though we believe that such events often reflect regions with large gene tree depths: in three of the four data sets, this measure actually increased slightly with the restriction, and in the fourth (CWBHM) it decreased slightly.

(9) We excluded CpG divergent sites from analysis because they are known to mutate at a higher rate than other sites in the genome.

(10) We excluded divergent sites with greater than two alleles across all five groups from analysis, because the analysis is specific to sites with a one-mutation history.

1. Patterson N, Richter DJ, Gnerre S, Lander ES, Reich D (2006) Genetic evidence for complex speciation of humans and chimpanzees. Nature 441: 1103-1108. [↑](#footnote-ref-2)
2. Benson G (1999) Tandem repeats finder: a program to analyze DNA sequences. Nucleic Acids Res 27: 573-580. [↑](#footnote-ref-3)
3. Cheng Z, Ventura M, She X, Khaitovich P, Graves T et al. (2005) A genome-wide comparison of recent chimpanzee and human segmental duplications. Nature 437: 88-93. [↑](#footnote-ref-4)
